# Insights into the Antibacterial Mechanism of Action of Chelating Agents by Selective Deprivation of Iron, Manganese, and Zinc

**DOI:** 10.1128/AEM.01641-21

**Published:** 2022-01-25

**Authors:** Joy R. Paterson, Marikka S. Beecroft, Raminder S. Mulla, Deenah Osman, Nancy L. Reeder, Justin A. Caserta, Tessa R. Young, Charles A. Pettigrew, Gareth E. Davies, J. A. Gareth Williams, Gary J. Sharples

**Affiliations:** a Department of Biosciences, Durham University, Durham, United Kingdom; b Department of Chemistry, Durham University, Durham, United Kingdom; c Procter and Gamble, Mason Business Center, Cincinnati, Ohio, USA; d Procter and Gamble Technical Centres Ltd., Reading, Berkshire, United Kingdom; University of Michigan-Ann Arbor

**Keywords:** chelating agents, metals, antibacterial agents, iron, manganese, zinc

## Abstract

Bacterial growth and proliferation can be restricted by limiting the availability of metal ions in their environment. Humans sequester iron, manganese, and zinc to help prevent infection by pathogens, a system termed nutritional immunity. Commercially used chelants have high binding affinities with a variety of metal ions, which may lead to antibacterial properties that mimic these innate immune processes. However, the modes of action of many of these chelating agents in bacterial growth inhibition and their selectivity in metal deprivation *in cellulo* remain ill-defined. We address this shortcoming by examining the effect of 11 chelators on Escherichia coli growth and their impact on the cellular concentration of five metals. The following four distinct effects were uncovered: (i) no apparent alteration in metal composition, (ii) depletion of manganese alongside reductions in iron and zinc levels, (iii) reduced zinc levels with a modest reduction in manganese, and (iv) reduced iron levels coupled with elevated manganese. These effects do not correlate with the absolute known chelant metal ion affinities in solution; however, for at least five chelators for which key data are available, they can be explained by differences in the relative affinity of chelants for each metal ion. The results reveal significant insights into the mechanism of growth inhibition by chelants, highlighting their potential as antibacterials and as tools to probe how bacteria tolerate selective metal deprivation.

**IMPORTANCE** Chelating agents are widely used in industry and consumer goods to control metal availability, with bacterial growth restriction as a secondary benefit for preservation. However, the antibacterial mechanism of action of chelants is largely unknown, particularly with respect to the impact on cellular metal concentrations. The work presented here uncovers distinct metal starvation effects imposed by different chelants on the model Gram-negative bacterium Escherichia coli. The chelators were studied both individually and in pairs, with the majority producing synergistic effects in combinations that maximize antibacterial hostility. The judicious selection of chelants based on contrasting cellular effects should enable reductions in the quantities of chelant required in numerous commercial products and presents opportunities to replace problematic chemistries with biodegradable alternatives.

## INTRODUCTION

Several transition metals are essential micronutrients for all organisms. An intricate balance of each has to be maintained to avoid deficiency or the toxic consequences of excess. Nutritional immunity, a component of the human innate immune system, makes use of the sequestration of metal ions in order to combat bacterial proliferation by starving such microorganisms of the metal ions they require (1, 2). The bioavailability of iron, manganese, and zinc, in particular, is severely constrained in the human body. Bacteria attempt to counteract this host-mediated metal starvation by upregulating metal selective importers and synthesizing and exporting their own chelators, such as enterobactin, to assist in metal uptake (2, 3).

Synthetic chelating agents form stable complexes with a variety of metal ions, and they have the potential to mimic the metal starvation and bacterial growth restriction conditions produced by nutritional immunity. Chelants are widely used in industry, with global consumption of aminopolycarboxylates (e.g., DTPA [diethylenetriaminepentaacetic acid], EDTA) alone estimated at 200,000 tons per annum at the beginning of the century (4). Myriad applications include water softening, effluent treatment, paper and textile manufacture, fertilizers, soil remediation, food processing, pharmaceuticals, medical detoxification, cosmetics and detergents, soaps, and disinfectants employed in both industrial and domestic settings (5, 6). In many cases, chelants function as potentiators that assist preservation and thus extend the shelf life of products (7–10). Despite their importance in product formulations, the antibacterial mechanism of action of chelating agents has received little attention in recent years, with current knowledge relying on studies concentrating on the consequences for bacterial outer membrane integrity (9). Experiments with the broad-spectrum chelating ligand EDTA suggest that it damages Escherichia coli by disrupting membrane permeability, possibly through the sequestration of Ca(II) and Mg(II) ions that stabilize the lipopolysaccharide (LPS) at the bacterial outer surface (9, 11–14). Treatment of E. coli with EDTA enhances susceptibility to various compounds, including amines (15) and antibiotics (16–19), consistent with interference with outer membrane permeability (20, 21). Similar observations have been made with other Gram negatives, including Pseudomonas aeruginosa (17, 22–25). Cell envelope damage by EDTA has been directly visualized by atomic force microscopy of both E. coli (26) and P. aeruginosa (27). Destabilization of artificial lipid membranes has also been reported as a consequence of EDTA exposure (28). Few reports have been published on the effect of any other chelants on bacteria, and none appear to have examined their impact on metal homeostasis.

In this study, we sought to probe the effects of several different chelating ligands on metal ion acquisition in the archetypal Gram-negative bacterium E. coli. The impetus for such a study arose from the use of chelants as bacteriostatic agents in a variety of consumer products and an eagerness to develop alternatives to ligands such as EDTA, which largely resist biodegradation (29). To this end, we have characterized the influence of 11 chelants on E. coli growth, individually and in combination, and determined their impact on total cellular metal ion concentrations. Our key objectives were to (i) identify the specific metals affected by chelant exposure and their contribution to bacterial growth restriction, (ii) assess chelators as potential probes for metal homeostasis that mimic nutritional immunity processes, (iii) explore the potential of combinations of chelating agents in antibacterial hostility, and (iv) to begin to rationalize such observations in relation to bacterial metallostasis.

## RESULTS AND DISCUSSION

### Chelant selection and inhibitory effects on E. coli growth.

**FIG 1:**
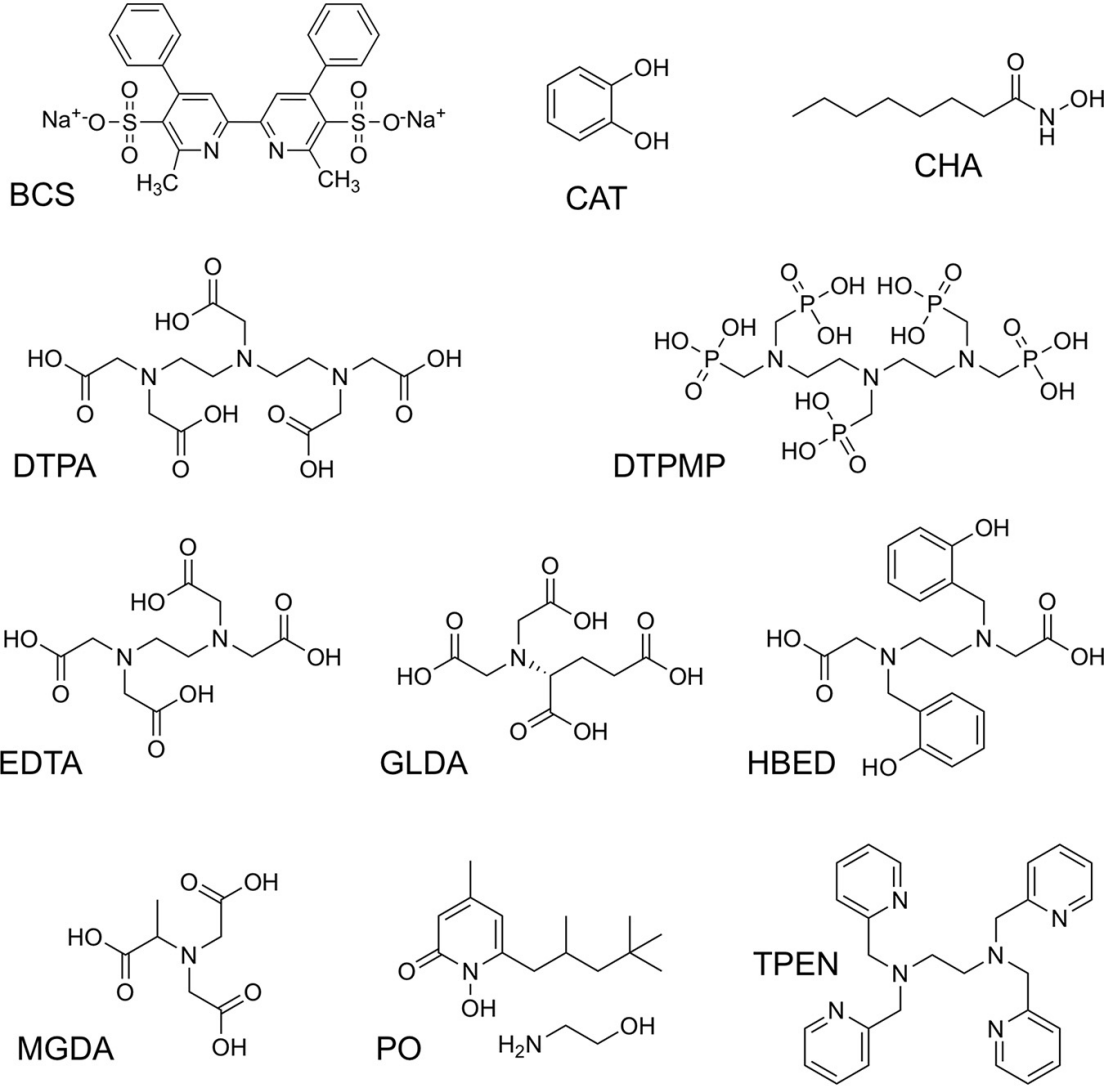
Structure of chelants selected for analysis. BCS, bathocuproine disulphonic acid; CAT, catechol; CHA, caprylhydroxamic acid; DTPA, diethylenetriaminepentaacetic acid; DTPMP, diethylenetriaminepentamethylene phosphonic acid; EDTA, ethylenediaminetetraacetic acid; GLDA, glutamic acid-*N*,*N*-diacetic acid; HBED, *N*,*N*-bis (2-hydroxybenzyl) ethylenediamine-*N*,*N*-diacetic acid; MGDA, methylglycinediacetic acid; PO, piroctone olamine; TPEN, *N*,*N*,*N*′,*N*′-tetrakis (2-pyridylmethyl) ethylenediamine. The most biodegradable isomer of GLDA (l-GLDA) is shown.

Eleven chelators were selected based on their known or predicted metal ion affinities (30–32) and differing chemical structures that might elicit a variety of complementary cellular effects (Fig. 1; Table S1 in the supplemental material). Most of the chelants are commonly known by their abbreviations rather than their full chemical names. The selection includes EDTA (hexadentate), its octadentate analogue DTPA, and closely related biodegradable aminocarboxylates GLDA (glutamic acid-*N*,*N*-diacetic acid) and MGDA (methylglycinediacetic acid), all of which are expected to bind a broad range of metal ions strongly, especially Fe(III). Metal ion affinities are quantified in terms of stability constants (association constants), namely, the equilibrium constant *K_a_* for the equilibrium M (metal ion) + L (ligand) ⇌ ML at a given pH, ionic strength, and temperature, typically expressed as log *K_a_*. Where available, log *K_a_* values for the selection of chelants used in this study for a number of biologically relevant divalent cations in combination with Fe(III) are listed in Table S1. The metal ion affinities of GLDA and MGDA (29) are lower than those of DTPA and EDTA (Table S1), indicating that higher concentrations may be required to chelate biologically relevant metal ions. DTPMP (diethylenetriaminepentamethylene phosphonic acid) has a similar nitrogenous backbone to DTPA but possesses five pendant phosphonates −P(O)(OH)_2_ instead of carboxylates −C(O)OH. HBED [*N*,*N*-bis (2-hydroxybenzyl) ethylenediamine-*N*,*N*-diacetic acid] is another aminocarboxylate, but it also incorporates phenolic units that favor binding to Fe(III) (31, 33). Catechol (CAT; a unit that occurs in enterobactin) has very high selectivity for Fe(III) *in vitro* (34), although its effective binding strength at pH 7 is attenuated due to competitive protonation. CHA (caprylhydroxamic acid) is a simple hydroxamate that resembles the constituent binding units of the siderophore desferrioxamine, which binds Fe(III) extraordinarily strongly (35). Piroctone (PO; the metal binding unit of piroctone ethanolamine) is a related cyclic hydroxamate. TPEN [*N*,*N*,*N*′,*N*′-tetrakis (2-pyridylmethyl) ethylenediamine] and BCS (bathocuproine disulphonic acid) are “softer” ligands that favor binding to late-transition metals such as Zn(II) (36) and Cu(I) (37), respectively.

**FIG 2:**
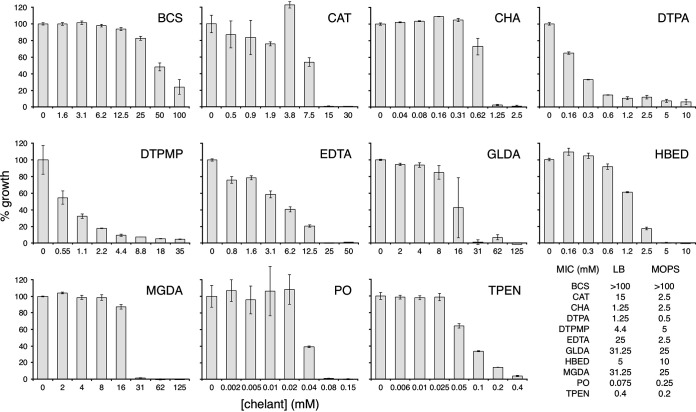
Effect of chelants on E. coli growth in LB. Bacteria were cultivated in LB (Lennox) media and mixed with appropriate 2-fold dilutions of each chelant and incubated with shaking for 16 h at 37°C. Results are the mean and standard deviation of an independent experiment performed in triplicate; a further two independent experiments performed in triplicate yielded similar results. MICs in mM are based on >90% growth inhibition, where achieved, as indicated at the bottom right of the figure; they were determined for LB and MOPS minimal media from 3 and 4 biological replicates, respectively.

The effect of each of the 11 chelants on bacterial growth was evaluated using 2-fold serial dilutions of each ligand (Fig. 2). The E. coli K-12 strain BW25113 was chosen to allow comparisons with deletion mutants from the Keio collection, a comprehensive set of single-gene knockout mutants (38). LB (Lennox) broth was selected as the growth medium, as it is widely used in cultivation of E. coli and offers good reproducibility. The provision of a rich growth medium with no inorganic nutrient restrictions (39) allowed an assessment of sensitivity to chelants when bacteria are in a robust physiological state. Bacterial growth was evaluated based on the absorbance at 600 or 650 nm (*A*_600_ or *A*_650_, respectively) after incubation with the chelant(s) for 16 h as in MIC determination assays (40). One of the chelants tested, BCS, failed to inhibit bacterial growth fully, even at the highest concentrations tested (Fig. 2). The dose-response curves with different chelants also varied, with several chelants (CAT, CHA, GLDA, MGDA, and PO) exerting little effect on growth until a particular threshold concentration was reached. Others (DTPA, DTPMP, and EDTA) resulted in higher susceptibility at low chelant concentrations and produced a correspondingly linear reduction in growth (Fig. 2). These different sensitivity profiles could be an indication of dual antibacterial effects, such as metal starvation coupled with membrane permeabilization, invoked previously as an explanation for the biphasic inhibition profile of EDTA with P. aeruginosa (8). DTPA and EDTA share similar molecular structures (Fig. 1) that may correspond to an analogous mechanism of growth inhibition. In most cases, high concentrations were required to achieve E. coli growth inhibition of >90%. To validate these findings, the experiments were repeated with the chemically defined MOPS (morpholinepropanesulfonic acid) minimal medium supplemented with glucose as the sole carbon source (41). In general, a similar pattern of effects was observed in comparison with the more complex LB broth (Fig. S1 in the supplemental material). The minor changes seen with BCS, HBED, and TPEN may reflect differences in the quantities or relative proportions of the metals present in each medium (see below). The MICs were also similar (Fig. 2), although 6-fold less CAT and 10-fold less EDTA were required to inhibit the growth of E. coli in the minimal medium relative to LB. The two chelants with highest efficacy in both media were PO and TPEN with MICs of 75 and 400 μM in LB and 250 and 200 μM in MOPS minimal medium, respectively (Fig. 2). PO activity is, however, ambiguous, owing to it being comprised of two components. We separated the piroctone from the ethanolamine and found that the former induced growth inhibition comparable to PO, whereas the latter was around 500-fold less active (Fig. S2). Thus, it is the piroctone fragment that is functionally active in bacterial growth reduction, and its activity can reasonably be attributed to its chelating ability.

The concentration of metal ions in the LB (Lennox) medium used was determined by inductively coupled plasma mass spectrometry (ICP-MS) (Fig. S3) to provide insight into availabilities prior to examining the effect of chelants on cellular metal content. The metal composition corresponds well with estimates from previous studies using LB (Miller) broth (Fig. S3) (42, 43). The metal content of MOPS minimal medium was also analyzed and found to contain 3.5-fold more magnesium and 2.4-fold more iron, but 19.5 times less calcium than LB (Lennox). Interestingly, the levels of zinc were below the threshold of detection (Fig. S3), although these low concentrations are not likely to be limiting for E. coli (44).

### Total cellular metal content of E. coli exposed to metal chelators.

**FIG 3:**
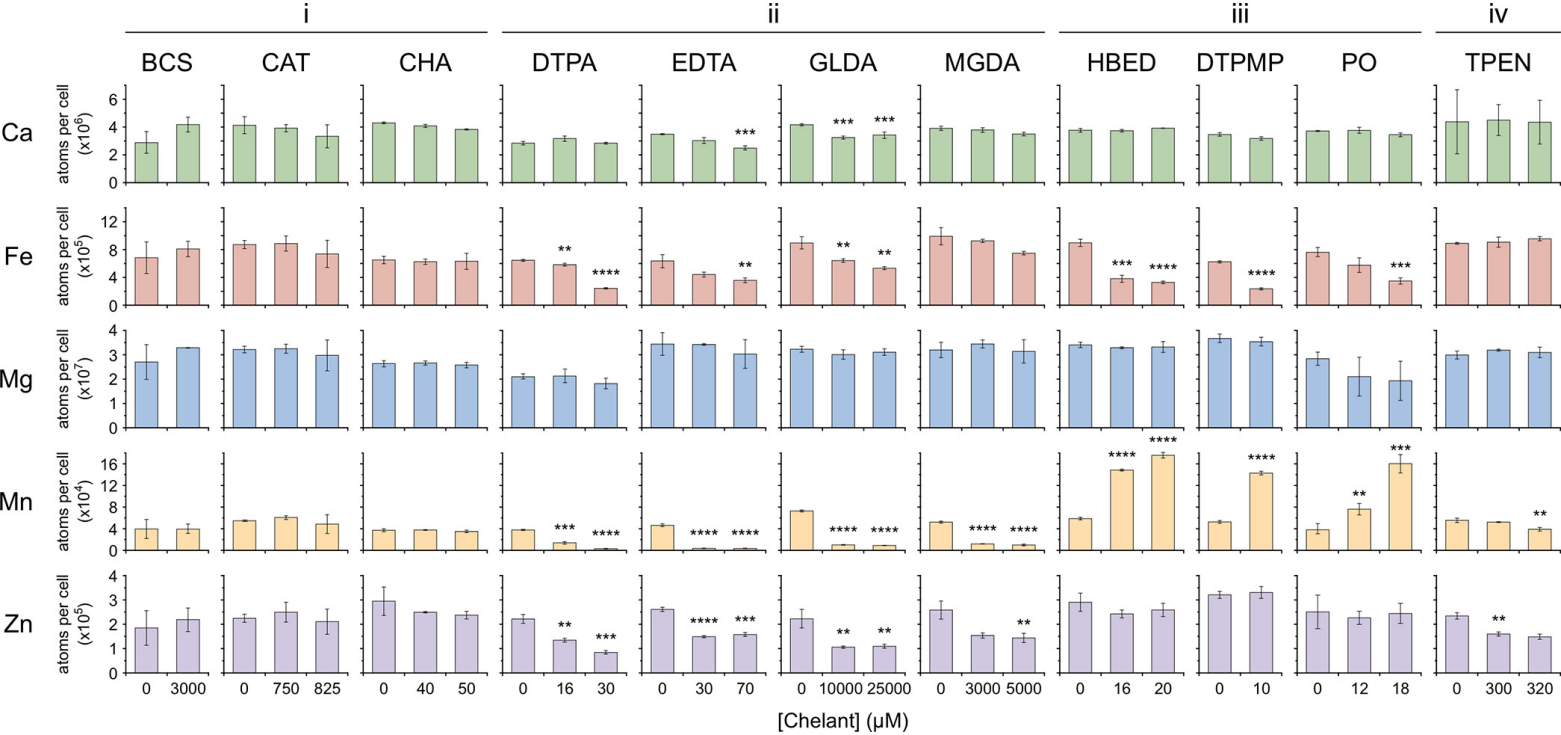
Effect of chelants on the metal composition of E. coli. Chelants are grouped according to the similarity in their effects on cellular metal concentration (i to iv) as described in the text. Selected results correspond to growth inhibition of 10 to 15% and the amount of each metal determined in atoms per cell using ICP-MS. BW25113 cells were grown in 50 ml of LB pH 7 to log phase in a shaking incubator (125 rpm) at 37°C. Data are the mean and standard deviation of 3 independent biological replicates (one-way analysis of variance [ANOVA] comparing each chelant concentration with the untreated control in each case; **, *P < *0.01; ***, *P < *0.001; ****, *P < *0.0001). Concentrations of each chelant are indicated below each set of graphs. The original data were determined over a range of chelant concentrations in most cases, and a full summary is provided in Table S2 in the supplemental material.

In order to probe the effect of chelants on cellular metal composition, we exposed E. coli to concentrations of each ligand that resulted in 10 to 15% growth inhibition in mid-log phase in LB (Lennox) media. Modest growth inhibition rates were chosen to avoid cellular damage that could potentially skew metal content measurements, owing to increased permeability or cell death. In addition, growth reductions at such low chelant concentrations can be reasonably correlated with cellular metal deprivation. It should be noted that chelating agents that associate with the envelope or reach the cytosol could sequester metals, but these cannot be differentiated from the bioavailable metals also present within the cell. Hence, any decreases detected in cellular metal content must be primarily caused by depletion of metals from the extracellular environment or from the bacterial exterior surface. The total number of calcium, iron, magnesium, manganese, and zinc ions in each cell was determined using ICP-MS. Copper was also measured, but its low concentration made determination less accurate and more prone to variation. Analysis of cobalt and nickel was not undertaken due to the extremely low levels present in E. coli. Cobalt is not required by E. coli (45), and nickel is only utilized by a small number of [NiFe] hydrogenase isozymes (46). Bacteria were grown in the presence of each chelant and harvested in mid-exponential phase, and the total cellular metal composition, expressed in atoms per cell, was determined relative to controls in the absence of the chelant (Fig. 3 and Table S2).

Four distinct categories of effect on cellular metal content were identified, primarily through differential effects on zinc, iron, and manganese concentrations (Fig. 3), and the results are discussed below according to these functional groupings. It is notable that cellular levels of calcium or magnesium were largely unaffected by exposure to each of the ligands.

### (i) No apparent effect on cellular metal content: BCS, CAT, and CHA.

The results with BCS, CAT, and CHA were unexpected in that they showed no significant impact on the metal composition of E. coli cells (Fig. 3; Table S2); these chelants also had no effect on copper levels, although the results with BCS were highly variable (Table S2). Either they act by a completely different mechanism to restrict bacterial growth (i.e., not involving the perturbation of metal availability), or, perhaps more likely, they sequester metals within the cell, making them inaccessible to the proteins that require them for functionality. This could potentially occur at the inner or outer membrane, the periplasm, or in the cytosol, depending on whether the chelator can traverse the cell wall barrier. It is suggested that chelant-membrane interaction might be more likely in these cases, based on the lipophilicity of these ligands, as reflected by estimations of their partition coefficients in their most likely ionization states at neutral pH. For example, the long hydrophobic tail and polar head in CHA could potentially insert into the outer membrane and thereby trap essential metals at the surface so they cannot gain access to the cell.

### (ii) Decreased manganese, iron and zinc: DTPA, EDTA, GLDA, and MGDA.

The principal effect of the azacarboxylate ligands DTPA, EDTA, GLDA, and MGDA at 10 to 15% growth inhibition is to deplete E. coli of manganese, with the reductions ranging from 5- to 15-fold relative to untreated controls (Fig. 3; Table S2). Zinc concentrations were also reduced at relatively low concentrations of each of these chelants (Fig. 3; Table S2). The total cellular content of iron was also lowered with DTPA, EDTA, and GLDA, but not significantly with MGDA (Fig. 3; Table S2). Small reductions in calcium levels were apparent with EDTA and GLDA. The preferential targeting of manganese is surprising given that these chelants would be expected to show a clear preference for iron based on log *K_a_* values (Table S1). There are a number of manganese-dependent enzymes in E. coli that could be rendered inactive by manganese starvation, including Mn-superoxide dismutase SodA (47), Mn-dependent ribonucleotide reductase NrdEF (48), and the heme biosynthetic enzyme coproporphyrinogen III oxidase HemF (49). Mismetallation of these enzymes (45, 50) and loss of the antioxidant properties of manganese could result in cells being more prone to damage by reactive oxygen species (51). However, low levels of manganese are not problematic for E. coli cells unless iron is scarce or they are exposed to hydrogen peroxide (52). Hence, the additional reductions in iron and zinc, alongside manganese depletion, likely impact on multiple metabolic systems and disrupt compensatory pathways for metal import (see below). We investigated this further by supplementing cultures with manganese chloride in the presence of EDTA (Fig. S4). Both EDTA and Mn(II) cause E. coli growth inhibition in a concentration-dependent manner (Fig. S4A and B). When EDTA and Mn(II) are mixed at different ratios, improved growth was observed (Fig. S4C and D), consistent with reversal of the cellular manganese deficiency. However, this response could simply be a consequence of EDTA-Mn(II) association in the medium, with the complexes formed moderating the adverse effects associated with EDTA metal sequestration. Supplementation of EDTA-treated P. aeruginosa and Salmonella enterica serovar Typhimurium cells with Ca(II) and Mg(II) has been previously reported (7, 17, 21, 53), with the positive effects attributed to either membrane stabilization or alleviation of the detrimental EDTA excess by chelant-metal binding.

### (iii) Decreased iron and elevated manganese: DTPMP, HBED, and PO.

DTPMP, HBED, and PO affect cells similarly to one another, reducing cellular iron concentration, coupled with a substantial increase in manganese (Fig. 3; Table S2). There was no significant change in levels of calcium, magnesium, or zinc (Fig. 3). E. coli cells are known to import manganese as a cellular response to iron starvation (45, 52). Manganese equivalents of iron-redox enzymes, e.g., Mn-superoxide dismutase (47, 54) and Mn-dependent ribonucleotide reductase (48), can substitute for iron-containing equivalents, while manganese can functionally substitute for iron in many mononuclear iron enzymes (45, 55). Iron and manganese metal homeostasis systems are linked via the ferric uptake regulator (Fur) and the proton-dependent manganese importer MntH (56). The E. coli Fur protein, when complexed with Fe(II), represses the expression of a suite of genes involved in iron uptake, metabolism, and bacterial virulence (57, 58). Thus, when iron levels are limiting, the affinity of Fur for its promoter sites is reduced, leading to upregulation of the iron homeostasis network. One such gene negatively regulated by Fur-Fe(II) is *mntH*, in accordance with the cellular response that switches to manganese import when iron is scarce (56, 59). The manganese superoxide dismutase (MnSOD) is similarly negatively regulated by Fur-Fe(II), whereas Fur-Fe(II) activates expression of iron superoxide dismutase, FeSOD (60, 61). Hence, as iron levels in the cell decrease, FeSOD levels decline just as MnSOD levels rise, concomitant with increased manganese uptake. The decreased levels of iron combined with increased manganese induced by DTPMP, HBED, and PO can reasonably be explained by bacterial adaptation to protect against iron starvation.

To further investigate the contrasting effects of PO and EDTA on cellular iron and manganese levels, we examined expression of the manganese importer by monitoring β-galactosidase activity from a reporter strain, SIP879, carrying an *mntH-lacZ* fusion (59). Interpretation of the experimental data is complicated by the fact that *mntH* is regulated by both MntR, the manganese regulator, and Fur, so we also tested a strain, SIP943, that lacks both *mntH* and *mntR* (59). MntR is a repressor of *mntH* promoter activity under manganese-replete conditions (59, 62). Treatment of SIP879 with PO induced expression of the *mntH-lacZ* promoter (Fig. S5A), a typical cellular response to iron starvation (59, 62). Similar expression levels between the *mntH-lacZ* and *mntH-lacZ mntR* strains exposed to PO (Fig. S5A) are also consistent with this being a Fur-mediated response to iron deprivation. Hence, iron restriction by PO would be expected to trigger manganese import by MntH, corroborating earlier findings (Fig. 3). The experiments were repeated with EDTA (Fig. S5B) as a representative of chelants that severely restrict cellular manganese concentration, alongside reductions in iron and zinc (Fig. 3). Interestingly, EDTA treatment resulted in activation of *mntH* (Fig. S5B), producing similar effects to PO and indicating that both chelants deprive cells of iron. As with PO, the levels of *mntH* expression were largely unaffected by the absence of *mntR* (Fig. S5B). EDTA has previously been reported to induce expression of *mntH* in both E. coli (59) and Salmonella (62). Thus, we can conclude that E. coli is subjected to iron starvation following exposure to EDTA. However, unlike the situation with PO, the cells are unable to switch to their regular recovery pathway because EDTA has also effectively removed access to manganese.

The effect of EDTA on bacterial growth following manganese chloride supplementation (Fig. S4) was revisited in experiments with the *mntH-lacZ* fusion (Fig. S5C). Inclusion of additional manganese to cells growing in LB broth did not induce expression from the *mntH* promoter as expected since MntR-mediated repression is only alleviated by manganese-limiting conditions (59, 62). Increased *mntH-lacZ* expression by EDTA was reduced by addition of manganese chloride, especially at equimolar concentrations (Fig. S5C). Similar results were obtained with SIP943, although lower levels of β-galactosidase activity were detected in response to EDTA in all of these experiments (Fig. S5C). While it is difficult to distinguish improvements in chelant tolerance due to either Mn(II) uptake by cells or removal of chelant toxicity by Mn(II) sequestration in the medium (Fig. S4), the absence of activation of *mntH-lacZ* when EDTA and Mn(II) are mixed in equal quantities argues in favor of the latter.

### (iv) Decreased zinc and manganese: TPEN.

The predominant effect of TPEN is on zinc concentration, consistent with its known affinity for Zn(II) (36), although, as with the other chelants, it will bind a range of metals (Table S1). At higher concentrations of TPEN, manganese levels are also slightly reduced and may contribute to growth inhibition by TPEN (Fig. 3; Table S2). However, at 300 μM TPEN, there is no reduction in Mn(II), whereas Zn(II) is reduced (1.5-fold). These results indicate that even relatively small reductions in cellular zinc levels may adversely affect E. coli, in keeping with earlier findings using zinc-depleted media (44). Microarray analysis of E. coli exposed to TPEN (63) links chelant exposure with increased expression of genes regulated by the zinc uptake regulator (Zur) (64) but also those controlled by Fur, implying that TPEN may not be entirely selective for zinc. TPEN is often referred to as a membrane-permeable chelator and has been reported to enter E. coli cells (36). Preferential removal of zinc from the extracellular environment can account for the reductions in cellular zinc observed here, but it is likely that intracellular zinc is also sequestered by TPEN and contributes to bacterial growth inhibition.

### Effect of chelant combinations on E. coli growth.

**FIG 4:**
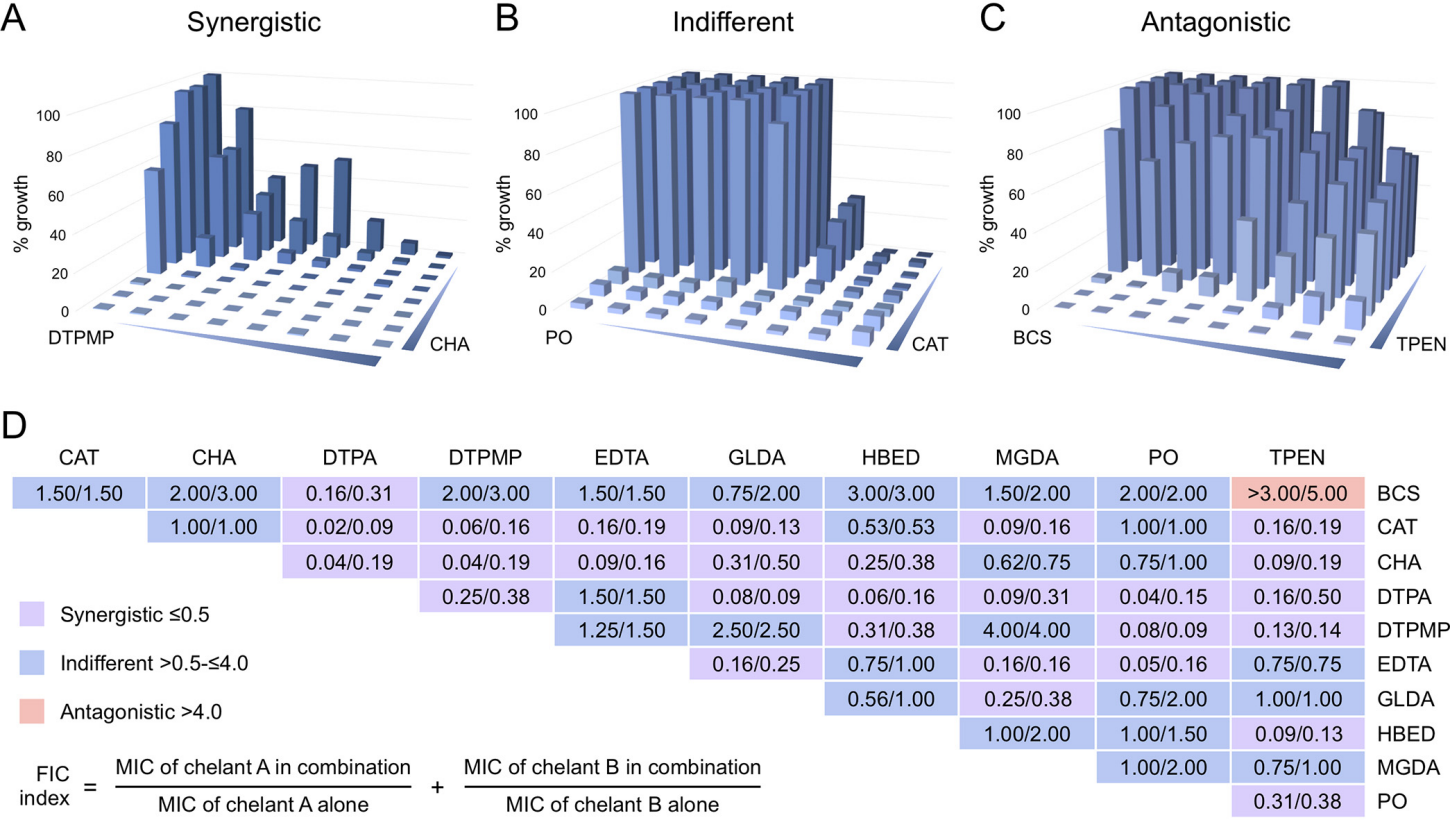
Chelant combinations analyzed by the checkerboard assay. Examples of synergistic (A), indifferent (B), and antagonistic (C) pairings for CHA-DTPMP, CAT-PO, and BCS-TPEN, respectively, are shown. (D) FICI values are shown for two independent experiments performed in triplicate for each chelant combination. The assay allows the calculation of an MIC for each chelant and, hence, the FIC, which provides a measure of the effect of the chelants in combination as synergistic, indifferent, or antagonistic. FIC index values were calculated based on the lowest concentration of each chelant in combination divided by the MIC for that chelant according to the formula shown. Twofold dilutions (as in an MIC) of chelants were performed in LB broth with E. coli BW25113 at 37°C with shaking at 37°C for 16 h. Additional controls for low levels of DMSO, ethanol, or water were included where relevant.

To gain further insight into the impact on bacterial metal restriction, pairs of chelants were tested based on the supposition that those affecting different metal uptake pathways should be synergistic when combined. The checkerboard, or two-dimensional, assay provides a simple way to evaluate inhibitory interactions between two soluble compounds and has been widely used to compare efficacies of different antibiotics in combination. The microdilution method used for our MIC assays was adapted with consideration of published protocols for the interpretation of checkerboard results (65, 66). The use of checkerboard assays is complicated with chelating agents because, in some cases, bacterial growth is never fully inhibited, unlike with many antibiotics. For instance, BCS at a maximal concentration of 100 mM only inhibits E. coli growth by 70 to 80% (Fig. 2). A percentage growth of <10% was chosen as a baseline for MICs, which are needed to calculate a fractional inhibitory concentration (FIC) index. For cases like BCS where <10% growth was not achieved, the maximum concentration of chelant provided the MIC and should be taken into account when assessing results obtained with BCS. Representative examples of synergistic, indifferent (or noninteracting), and antagonistic pairings from our studies are illustrated in Fig. 4A to C. Overall 55 chelant pairings were tested and FIC indices determined (Fig. 4D), revealing 1 antagonistic, 26 indifferent, and 28 synergistic combinations by selecting the lowest possible combination of each chelant in cumulative calculations (Data Set S1). Considerably fewer synergistic pairings, only 5 (plus 8 mixed synergistic/indifferent outcomes), were obtained using an average FIC method, although that is not surprising, as such an approach employs much stricter criteria for assigning synergy (67) (Fig. S6). Synergistic, indifferent, and antagonistic pairings are listed according to their effect on metal content to facilitate comparisons between groups (Fig. S7). DTPA yielded the highest number of synergistic pairings, with 9 partners (Fig. 4 and Fig. S7). BCS produced the lowest number, displaying synergism only with DTPA (Fig. S7), perhaps because of its limited capacity to fully inhibit bacterial growth (Fig. 2 and Data Set S1).

### Comparison of checkerboard and metal composition data.

We predicted that chelant categories that cause similar effects on cellular metal levels would produce indifferent outcomes when combined. Conversely, those with dissimilar effects on metal composition might be expected to yield synergistic results. To some extent, this proved to be the case, but there were notable exceptions (Fig. 4 and Fig. S7). Although the majority of the synergistic pairs do indeed match complementary categories of metal deprivation, there are 7 examples (DTPA-GLDA, DTPA-MGDA, DTPMP-HBED, DTPMP-PO, EDTA-GLDA, EDTA-MGDA, and GLDA-MGDA) where chelants individually induce analogous cellular responses to metals yet produce synergistic effects in combination. There are also multiple examples of chelants from the different metal effect categories defined earlier that show indifference (e.g., DTPMP-EDTA, GLDA-HBED, and MGDA-TPEN). The results suggest that there are several different ways that chelants function in depriving cells of metals, even for those that appear to have the same overall effect. Preferential removal of metal either from the media or at the bacterial surface as a function of chelant structure may account for some of these differences. Alternatively, there may be effects produced by chelant-metal association at membranes or in the cytosol that influence metal accessibility. It is interesting to note that CAT and CHA display an identical pattern of synergistic and indifferent outcomes with 7 other chelants and are also indifferent with each other (Fig. S7). These findings suggest that CAT and CHA are functionally equivalent in depriving cells of the same subset of metals despite their dissimilar structures (Fig. 1). This is informative since neither of these chelants appeared to affect total cellular metal content (Fig. 3).

Phenolic compounds, such as CAT, are known to form brown complexes with Fe(III) with absorbance between 380 and 800 nm (68), and this was apparent when CAT was mixed with media in the presence (Fig. S8A) or absence (Fig. S8B) of bacteria. Different chelant combinations with CAT exacerbated or alleviated the formation of these colored complexes (Fig. S8A). Those chelants that deprive cells of iron (Fig. 3), such as HBED and PO, appear to reduce the formation of this complex as judged by a loss of color. In contrast, those predominantly affecting manganese, such as EDTA and GLDA, promote the formation of the dark brown color (Fig. S8A). The comparatively high concentrations of these chelants, coupled with their relative affinities for different metals, likely serve to remove competing metals from the media, thereby making iron more available for sequestration by CAT. Depending on their commercial application, certain chelant combinations might be best avoided because of the production of pigment, although at lower concentrations, this may not be problematic.

TPEN is synergistic with all but four chelants (BCS, EDTA, GLDA, and MGDA), indicating that reductions in cellular zinc levels might be highly effective as a means of restricting bacterial growth when combined with chelants that primarily limit the availability of other metals. Four chelant pairings (DTPA-DTPMP, DTPA-HBED, DTPA-PO, and EDTA-PO) that mainly reduce manganese or iron levels produce synergistic outcomes, although many more chelants from these two categories do not (Fig. 4 and Fig. S7). Membrane damage associated with EDTA (9, 14, 20), and potentially with the structurally related DTPA, may serve to drive partner chelants across the bacterial outer membrane and allow targeting of the periplasm or cytosol. This might account for DTPA-GLDA and EDTA-GLDA synergism, despite all three having similar effects on deprivation of cellular manganese, zinc, and iron. In addition, some chelants (e.g., HBED, PO) are somewhat lipophilic and could associate better with membranes, particularly if the LPS layer is damaged. This fits with the iron binding ligands PO and HBED being synergistic with the hydrophilic DTPMP, another iron chelator (Fig. 4 and Fig. S7). Hence, the effect of metal starvation, coupled with membrane damage or penetration, could be instrumental in the bacterial growth restriction phenotype seen with these chelating agents.

### Analysis of bacterial metal content with chelants in combination.

**FIG 5:**
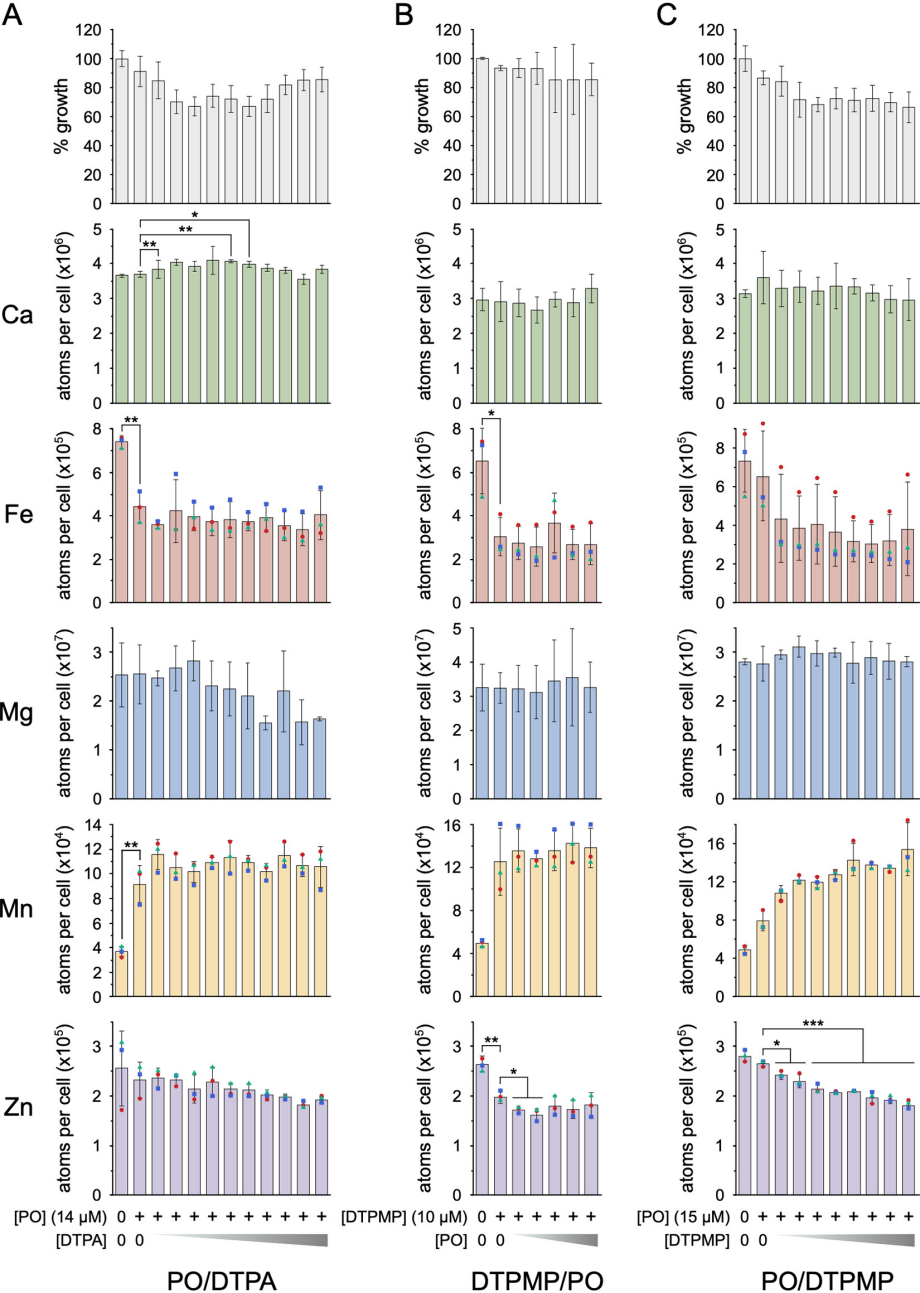
Effect of selected chelant combinations on total cellular metal content. (A) Combination of 14 μM PO with 2, 4, 6, 7, 7.5, 8, 8.5, 9.5, and 10 μM DTPA. (B) Combination of 10 μM DTPMP with 12, 13, 14, 15, and 16 μM PO. (C) Combination of 15 μM PO with 1.25, 2.5, 3.75, 5, 6.25, 7.5, 8.75, and 10 μM DTPMP. Concentrations of each chelant used are indicated below each set of graphs. Results correspond to growth inhibition of 10 to 30% (gray bars in the topmost graphs) and the amount of each metal determined in atoms per cell using ICP-MS. BW25113 cells were grown in 50 ml of LB to log phase in a shaking incubator at 37°C. Data are the mean and standard deviation of 3 independent biological replicates (one-way ANOVA comparing each chelant concentration with the untreated control in each case or between single chelant treatment with addition of a second chelant as indicated; *, *P < *0.05; **, *P < *0.01; ***, *P < *0.001). Although some experiments were subject to variability, there were consistent trends with Fe, Mn, and Zn levels in each of the three independent experiments in some cases; for these metal ions, the data points are highlighted as differently colored symbols to show the pattern of each of the three replicates.

To further understand how chelant combinations exert synergistic effects, we selected two synergistic pairs, DTPA-PO and DTPMP-PO, which show distinct effects on cellular metal composition. A fixed concentration of the first chelant producing ∼10% bacterial growth inhibition was employed with increasing amounts of the second chelant to produce a 10 to 30% final growth restriction. As before, the cellular levels of calcium, iron, magnesium, manganese, and zinc were determined using ICP-MS (Fig. 5). Selected results showing the proportional change in metal content from experiments with PO in combination with either DTPA or DTPMP are also shown in Table S3.

DTPA and PO have a radically different impact on the metal composition of E. coli and function as a highly synergistic pair (Fig. 4); DTPA depletes cells of manganese, alongside reductions in iron and zinc, whereas PO increases manganese in response to iron limitation (Fig. 3). We predicted that synergy might be due to DTPA preventing the influx of manganese induced by PO. However, the results showed that the effect of PO seems to dominate over that of DTPA, yielding results similar to PO alone (Fig. 5A; Table S3). Modest increases in calcium were evident at a few concentrations of both chelants (Fig. 5A), but there were no significant changes in other metals comparing PO alone with the PO-DTPA combination. As suggested above, the potential influence of DTPA on membrane integrity could exacerbate the activity of the lipophilic PO.

In contrast to the DTPA-PO pairing, DTPMP and PO behave similarly in reducing levels of iron and increasing manganese yet display a synergistic effect on E. coli growth where an indifferent response was anticipated. There was little change in metal levels between the effect of DTPMP alone and samples that combined DTPMP with increasing amounts of PO, apart from some reduction in zinc at lower PO concentrations (Fig. 5B; Table S3). It should be noted that a small but significant reduction in zinc was evident with DTPMP (Fig. 5B) that was not detected previously with this chelant (Fig. 3). To probe this further, the reciprocal experiment was performed using a fixed concentration of PO and titration of DTPMP (Fig. 5C; Table S3). In this case, a significant reduction in zinc was evident at all concentrations of both chelants relative to PO alone. Although the results are not statistically different due to variability in the data sets, there was also a consistent decrease in iron and increase in manganese when the chelants were combined (Fig. 5C, compare the symbols for each data set). These results are in keeping with DTPMP and PO, producing the same effects on cellular levels of manganese and iron, but an additional reduction in zinc when combined. The latter effect may be responsible for the synergism observed between these two chelants (Fig. 4).

### Effect of PO on the growth of E. coli mutants from the Keio collection.

**FIG 6:**
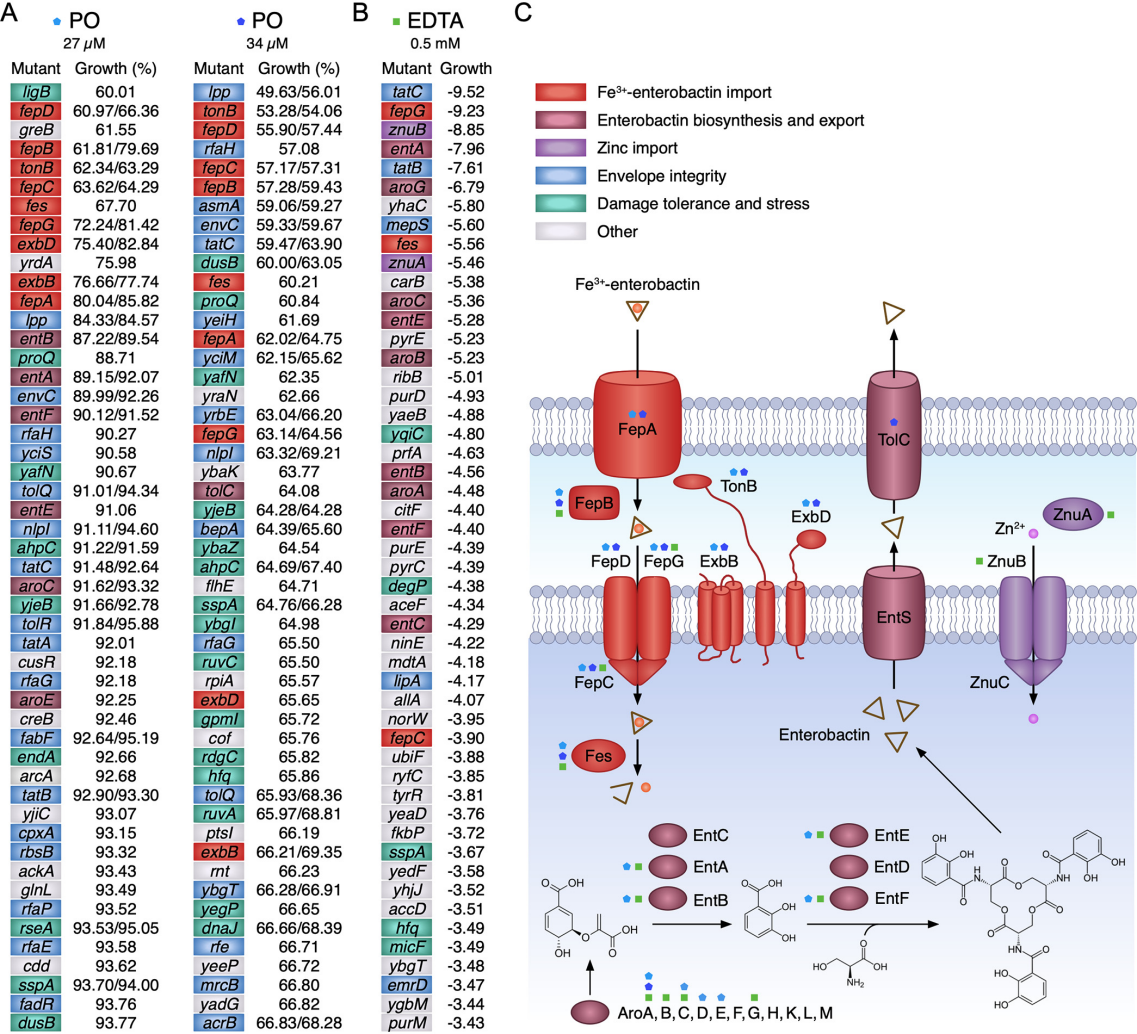
Effect of PO on the growth of E. coli mutants from the Keio collection. (A) The duplicate set of 3,985 Keio library mutants (38), 7,970 strains in total, were grown in LB media at 37°C with 27 or 34 μM PO for 16 h. Percentage growth was compared to untreated controls for each strain, and the top 50 slowest-growing mutants are shown (see Data Set S2 in the supplemental material). Where two percentages under each condition are shown, these correspond to the presence of both duplicates from the Keio collection in the top 200 slowest-growing mutants identified in the screen. Each mutant is color-coded based on the functional grouping assigned for each gene with the key shown in panel C. (B) The 50 slowest-growing mutants identified from the Keio phenotypic screen using EDTA (69) are shown to facilitate comparisons with PO. The more negative pixel score values correspond to the poorest colony growth on agar plates supplemented with 0.5 mM EDTA. (C) Ferric enterobactin synthesis and export and import system of E. coli. Key proteins involved in each part of the iron uptake system are color-coded according to their roles (71, 73). AroA-M proteins are involved in the biosynthesis of chorismate that is converted by EntABC to 2,3-dihydroxybenzoic acid (DHB). EntDEF catalyze DHB and l-serine linkage and ultimate assembly into enterobactin (71), which is exported to the extracellular environment by EntS and TolC (82). The ferric-enterobactin complex is recovered by association with the outer membrane receptor FepA. The TonB/ExbBD complex provides energy from the proton motive force to mediate release of the Fe(III)-enterobactin complex from FepA, facilitated by FepB, and delivery to the FepCDG ABC family, ATP-dependent inner membrane permease (71). Upon reaching the cytosol, Fe(III) is released from the siderophore by the Fes esterase (83). Another ABC family transporter, ZnuABC, transports Zn(II) across the inner membrane (72, 84). Outer and inner membranes are depicted as lipid bilayers with the lower portion of the diagram shaded in blue to represent the cytosol. Where substantially reduced growth is associated with mutation of key ferric-siderophore synthesis and transport components, these are indicated with cyan and blue (PO) and green (EDTA) symbols.

To provide insight into the gene products important for tolerating exposure to chelants, we next selected one of the iron chelators, PO, in a screen of the E. coli Keio collection of single-gene deletions to identify mutants with increased susceptibility. The duplicate set of the Keio collection of 3,985 mutants (7,970 strains in total) was cultivated in 96-well plates in LB media in the presence of low levels of PO at 27 and 34 μM. The growth of each strain exposed to PO relative to untreated controls was determined after overnight incubation and the most sensitive mutants identified (Fig. 6A; Data Set S2). The influence of EDTA on E. coli growth has previously been analyzed by inoculating the Keio collection mutants onto LB agar plates (69), and this facilitated comparisons with our data on PO (Fig. 6B). The Keio screen with PO highlighted the importance of genes involved in iron-siderophore uptake for PO tolerance (Fig. 6C). Mutants affecting enterobactin synthesis (Aro, Ent), export (TolC), and import (FepA-G, ExbBD-TonB, and Fes) were among those with the most substantial growth reductions relative to the control following PO exposure (Fig. 6A and C). Deletion mutants affecting envelope integrity, efflux pumps, damage tolerance, and stress responses also showed sensitivities to PO (Fig. 6A), potentially indicating that PO can more readily gain access to the periplasm or cytosol in these strains and thereby affect growth. Some similarities in growth behavior with EDTA (69) were observed with a similar subset of genes involved in enterobactin-iron uptake and membrane integrity affected. However, unlike PO, mutants defective in components of the Znu zinc uptake system showed impaired growth when exposed to EDTA (Fig. 6B).

A small number of mutants displayed improved growth relative to untreated controls when PO was incorporated in the growth medium (Table S4). Several of these mutants display better growth at both PO concentrations, suggesting that their deletion does correspond to a genuine improvement in growth. These mutants correspond to genes linked to regulatory pathways, metabolic processes, and repair of oxidative damage. However, the largest group of mutants affected are those engaged in flagellar biosynthesis, of which 26 *fli*, *flg*, and *flh* genes occur in the 200 mutants that show the most enhanced growth at both PO concentrations (Table S4). This may represent an alleviation of the substantial energy cost involved in flagellum assembly and operation (70) during the iron limitation imposed by PO. Significantly, flagellar gene-deficient mutants do not exhibit the most enhanced growth of the Keio mutant strains under low-iron conditions using MOPS media (69), suggesting that PO either exerts additional detrimental effects or targets iron depletion with a different cellular specificity.

### Effect of PO, EDTA, and DTPMP on the growth of selected E. coli Keio collection mutants.

**FIG 7:**
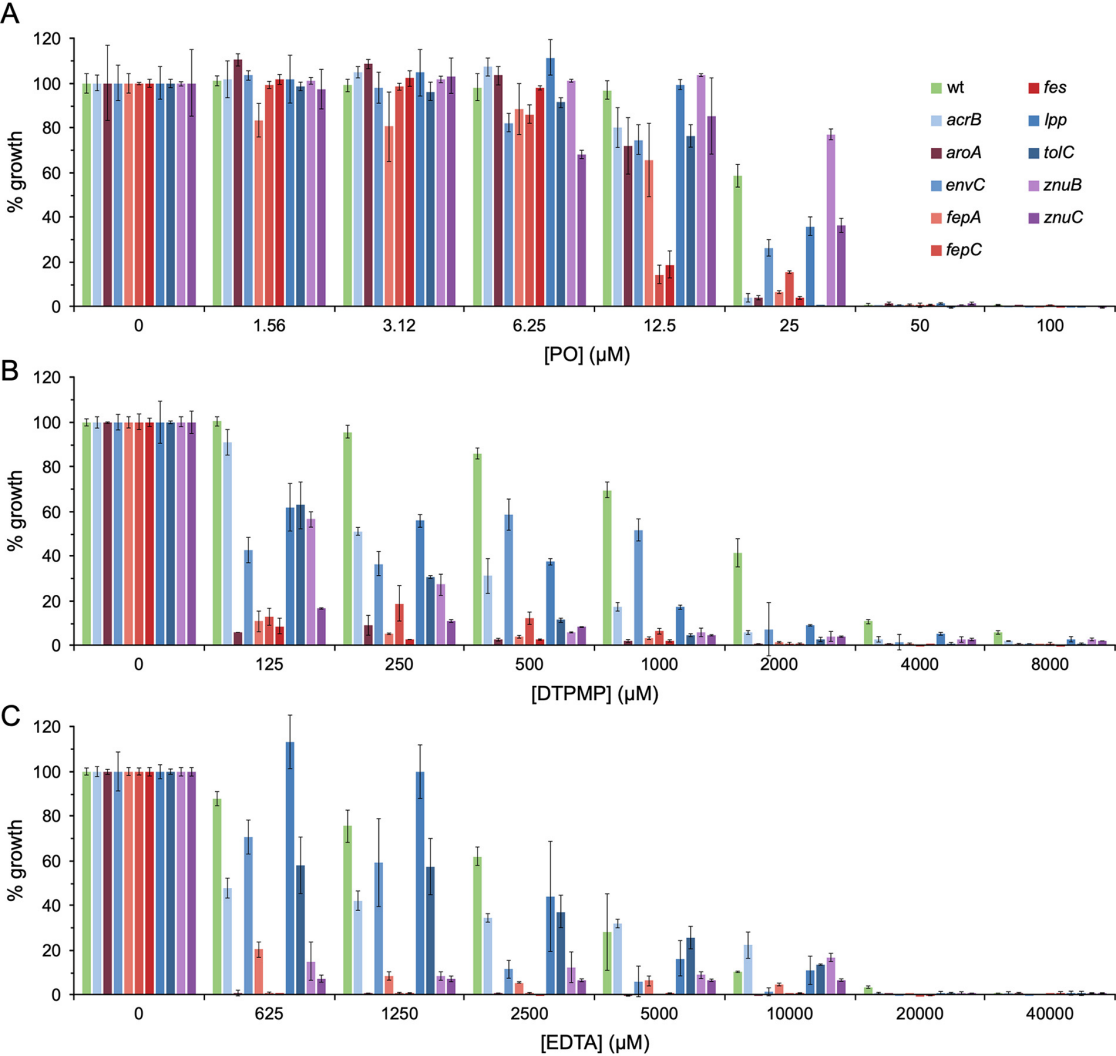
Selected E. coli mutant sensitivity to PO (A), DTPMP (B), and EDTA (C). Bacteria were incubated with a 2-fold serial dilution of each chelant in 100 μl of LB media and incubated with shaking for 16 h at 37°C. Absorbance at 600 nm was recorded and the percentage growth calculated for each strain. Data represent the mean and standard deviation of an independent experiment performed in triplicate. A second biological repeat was performed, and a similar pattern of susceptibility was observed.

To validate the findings with the Keio screen, we selected a range of the most PO-susceptible mutants and others deficient in related iron, manganese, and zinc uptake pathways for further testing. All of the mutants affecting enterobactin biosynthesis or uptake (*aroA*, *fepA*, *fepC*, and *fes*) (71) exhibited substantially reduced growth relative to the wild type (WT) following exposure to PO (Fig. 7A), consistent with the importance of iron acquisition for tolerance of this chelant. Interestingly, a corresponding sensitivity was not found with *fepB* and *fepD* mutants (Fig. S9A). Several strains lacking integral membrane proteins involved in drug export and envelope integrity (*acrB*, *envC*, and *tolC*) also showed some increased susceptibility as in the Keio screen with PO (Fig. 7A). Two mutants, *znuB* and *znuC*, affecting zinc import (72) behaved similarly to the WT as expected. Mutants affecting components of the Fe(III)-citrate (*fecA*, *fecB*, *fecD*, and *fecE*) and Fe(III)-hydroxamate (*fhuF*) systems (73) were generally no more susceptible to PO. Similarly, mutants involved in cysteine (*cysE*) and histidine (*hisI*) biosynthesis that are highly sensitive to iron starvation (69) showed no increased susceptibility to PO (Fig. S9A).

The same strains were also examined for their susceptibility to DTPMP and EDTA (Fig. 7B and C), the latter allowing comparisons with published data (69) that were conducted on solid rather than liquid media. As with PO, defects in the enterobactin pathway (*aroA*, *fepA*, *fepC*, and *fes*) produced the highest sensitivity to these two chelants, underlining the necessity of this route of iron acquisition for bacterial growth and defense against these chelants. In contrast to PO, the other ferric iron import pathway mutants also showed increased susceptibility, especially with DTPMP (Fig. S9B and C). Reduced growth following chelant exposure was apparent with mutants affecting membrane integrity functions.

Mutants in the *znuB* and *znuC* zinc import system were much more sensitive to DTPMP and EDTA (Fig. 7B) than PO (Fig. 7A), suggesting that reductions in cellular zinc—either due to mutation or sequestration by a ligand such as TPEN—increase chelant vulnerability. DTPMP-treated cells display low levels of iron and elevated concentrations of manganese (Fig. 3), however, small reductions in zinc were also apparent, especially when combined with PO (Fig. 5B). The enhanced susceptibility of *znu* mutants to DTPMP but not PO indicates that these two chelants do not behave precisely in the same way and that additional effects on zinc may account for their synergistic behavior (Fig. 4). Deletion of the manganese importer, MntH, did not result in decreased growth following EDTA exposure (Fig. S9C), in agreement with previous studies (69). In repeat assays, growth was actually improved following EDTA treatment in an *mntH* strain (Fig. S10). Although it is not clear why growth would be improved in the absence of *mntH*, these observations are consistent with combined reductions in iron, manganese, and zinc, rather than manganese alone, being important for bacterial growth inhibition by EDTA.

**Conclusions.** Using E. coli as a model organism, the specific metals affected by a selection of chelating agents have been identified and their impact on bacterial growth and metal deprivation evaluated. The cellular concentrations of calcium, iron, magnesium, manganese, and zinc were determined for 11 chelators with differing structures and metal ion selectivities. Four categories of chelants with distinct effects on metal depletion were identified.

BCS, CHA, and CAT do not appear to alter the levels of any of the metals tested, although it is possible that they trap particular metals, potentially at the cell surface, and thus prevent metals from accessing the cell. Hence, the metals would remain associated with the cell but would be unavailable to essential intracellular enzymes. Of these three, CHA and CAT appear to be functionally equivalent, as judged by their similar behavior in combination with other chelants.

DTPA, EDTA, GLDA, and MGDA all produce a dramatic decrease in cellular manganese, combined with lesser reductions in both iron and zinc. Iron and zinc limitation could well be the principal factor in bacterial growth inhibition with these chelants since E. coli mutants with defects in uptake pathways for these metals (e.g., *fepA*, *fes*, and *znuB*) are more sensitive to EDTA (Fig. 6 and 7) (69). That manganese has only a secondary effect, perhaps at the cell surface, fits with the improved growth of an EDTA-treated *mntH* mutant (Fig. S10), which lacks the MntH manganese transporter that would boost cytosolic levels of Mn(II) (55). Examination of *mntH* promoter activity (Fig. S5) confirmed that EDTA starves cells of iron, but is also likely to prevent manganese import by sequestration, making this route of tolerance ineffective. EDTA, and potentially DTPA, has known detrimental effects on outer membrane integrity (9, 27), meaning that a combination of metal starvation and membrane damage likely contributes to its antibacterial mechanism of action. It is feasible that stripping of manganese from a primary location at the bacterial surface is responsible for the injurious effects on membrane stability. The cellular location of manganese has yet to be established (55), although in the Gram-positive Bacillus subtilis, Mn(II) does appear to be associated with the cell wall (74).

Exposure of E. coli to DTPMP, HBED, and PO causes a reduction in iron and an influx of manganese; the triggering of manganese import is a known cellular defense response to iron starvation (45, 52), in keeping with these ligands being Fe(III) chelators. Experiments with combinations of these three chelants, however, suggest that they are not functionally equivalent and that their cellular targets may differ. Cells deficient in the zinc (Znu) uptake and ferric-citrate (Fec) pathways are hypersensitive to DTPMP but not PO (Fig. 7 and Fig. S9), and reductions in cellular zinc levels were apparent with DTPMP, especially when mixed with PO (Fig. 5). As with DTPA, EDTA, GLDA, and MGDA, the potential for membrane penetration or damage may account for the differing interactions observed.

**FIG 8:**
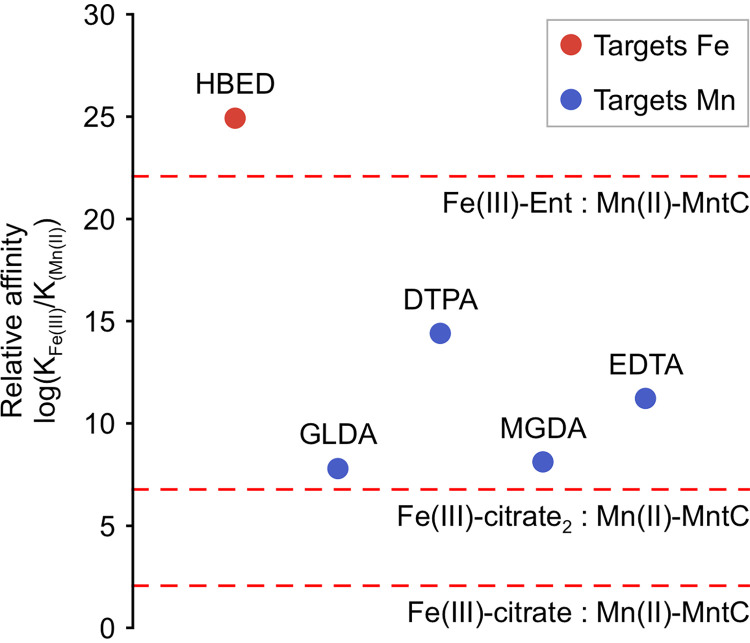
Relative Fe(III) and Mn(II) affinities of chelants which primarily restrict either Fe(III) (in red) or Mn(II) (in blue) accumulation in cells (judged by percent reduction of metal content in chelant-treated cells compared to untreated controls). The relative metal affinities of selected components of uptake systems for iron or manganese at the cell surface are shown by the red dotted lines. The association constants of Fe(III)-enterobactin and Fe(III)-citrate at pH 7.0 were calculated using reported pH-independent affinities of ligands and pK*_a_* values of coordinating atoms (85–87), the Mn(II) affinity of S. aureus MntC (solute binding protein) was used in the absence of a known affinity of the E. coli manganese transporter MntH (88), and a “pseudoaffinity” of the Fe(III)(citrate)_2_ complex was derived from the calculated pFe^3+^ at pH 7.4 when [Fe(III)]_total_ was equal to 1 μM and [citrate]_total_ was equal to 100 μM (75).

Why might certain chelants, such as EDTA and DTPA, deplete cells of manganese considerably more than iron? Affinities for both Fe(III) and Mn(II) are known for five of the chelants which interfere with the accumulation of these metals, namely, DTPA, EDTA, GLDA, HBED, and MGDA (Table S1). Figure 8 shows their relative affinities {as log[*K*_Fe(III)_/*K*_Mn(II)_] where *K*_metal_ corresponds to association constants *K_a_*} along with comparative estimated values for uptake systems for these two metals: note that an available *K*_Mn(II)_ from S. aureus MntC has been used in the absence of a measured value for E. coli MntH, and a pseudo-*K_a_* for Fe(III)-citrate_2_ was simulated for defined total Fe(III) and citrate concentrations (1 μM and 100 μM, respectively) (75). Importantly, only log[*K*_Fe(III)_/*K*_Mn(II)_] for HBED exceeds estimated values for all uptake systems (Fig. 8), and of the five chelants, only HBED impairs the uptake of iron and not manganese (Fig. 3). Thus, even if two chelants show the tightest affinity for the same metal, their relative affinities (for different metals) can drastically alter their impact on cellular metal acquisition systems. This preliminary analysis suggests that it may be possible to model bacterial responses to chelants based on relative metal affinities and by measuring *K_a_* for all uptake systems for all metals to predict cellular responses to chelants.

As outlined above, analysis of the cellular metal selectivity of the chelants tested allowed the identification of chelants with high specificity for iron, manganese, and zinc that could serve as mimics of nutritional immunity and as tools to probe bacterial metal homeostasis. Those with specificity for zinc and iron offer clear value, although those affecting manganese may exhibit too broad a range of metal target. There is considerable potential to exploit two, or even three, chelants to restrict bacterial growth in a range of consumer, industrial, and health care settings. For example, CHA, EDTA, and PO acting by different mechanisms could prove a potent antibacterial mixture. Chelators could be deployed in combination with antibiotics for wound care and other therapeutic applications, especially as they are implicated in disrupting biofilm formation (27, 76, 77). Metal toxicity could also be harnessed in the presence of chelants that selectively restrict availability of iron, manganese, and zinc to mimic the killing achieved in phagocytic vesicles (78). Modeling bacterial uptake of metals will assist in identifying the specificity of molecules for manipulating metal acquisition. Affinity ratios can identify which chelants preferentially interfere with which metals for uptake as exemplified by log[*K*_Fe(III)_/*K*_Mn(II)_] values for the iron-selective chelator HBED (24.9), which exceeds values estimated for Fe(III) versus Mn(II) uptake (22.1) and exceeds values for DTPA, GLDA, EDTA, and MGDA (≤14.4), which preferentially target manganese.

The results from this study show that, in most cases, it is challenging to predict, especially from available empirical metal ion affinity data, which combinations are likely to be most effective (79). However, we now have a much clearer understanding of the metals affected and indications that the cellular sites of metal sequestration may differ between them. Significantly, a large number of synergistic antibacterial chelant combinations have been identified that could be incorporated into products where their preservation properties are desirable. New formulations can be manufactured that reduce the quantities of chelants required and integrate biodegradable alternatives (e.g., GLDA-MGDA) with major benefits for sustainability and environmental compatibility. Further work is needed to rationalize our predictive capabilities with chelating agents and define precisely (i) the localization of chelants within cells, (ii) robust metal ion affinities for chelators to multiple metal ions *in vitro*, (iii) how these affinities compare with the availabilities (buffered concentration-free energies) of the elements at their respective locations (80), and (iv) whether bacterial species with different cell wall structures and metal uptake strategies exhibit similar cellular responses.

## MATERIALS AND METHODS

### Bacterial growth inhibition by chelants.

Chelating agents were obtained commercially and are listed in Table S5 in the supplemental material. Most chelants were soluble in water, but CHA, HBED, and PO were resuspended in dimethyl sulfoxide (DMSO) and TPEN in ethanol. Appropriate vehicle controls were performed in parallel for all growth experiments involving these chelants. E. coli K-12 BW25113 [*rrnB3* Δ*lacZ4787 hsdR514* Δ(*araBAD*)*567* Δ(*rhaBAD*)*568 rph-1*] and deletion-insertion derivatives from the Keio collection (38) were used in this study. For microdilution MIC assays, E. coli cultures were grown in LB media (Lennox; Sigma-Aldrich) or MOPS minimal media (Teknova Inc.) in an orbital shaker (Stuart) at 37°C to an *A*_650_ of 0.07, equivalent to a 0.5 MacFarland standard (240 μM BaCl_2_ in 0.18 M H_2_SO_4_ aq.) and diluted 10-fold in LB broth for use as an inoculum (65). The diluted culture (50 μl, 5 × 10^6^ CFU/ml) was then transferred into a 96-well, round-bottomed microtiter plate (Sarstedt). Chelants from stock samples, prepared in water, DMSO, or ethanol, were diluted to yield a 2-fold series in LB broth and 50 μl mixed with the diluted inoculum. Plates were incubated at 37°C with shaking at 130 rpm for 16 h and absorbance (*A*_600_ or *A*_650_) monitored on a Spectrostar Nano plate reader. MICs were defined as the minimum concentration of chelant needed to inhibit growth by >90% relative to controls.

Checkerboard assays were performed to assess the effect of chelants in combination. Stock solutions and inoculum were prepared as for MIC experiments. One chelator was applied in decreasing concentrations horizontally across the 96-well microtiter plate, while the second chelator was added in decreasing concentrations vertically to create the checkerboard (Data Set S1). A fractional inhibitory concentration index (FICI) was defined as the minimum concentration of chelant needed to inhibit growth by >90% individually and in combination, and FICI values were interpreted as synergistic (≤0.5), indifferent (>0.5 to 4.0), or antagonistic (>4) based on published methods (66, 67) and according to the formula shown in Fig. 4.

### Isolation of piroctone from piroctone olamine.

PO was dissolved in the minimum amount of methanol prior to the addition of 1 M HCl (until pH 1 was reached). The mixture was then transferred to a separating funnel, diluted with either dichloromethane (DCM) or chloroform, and the organic layer collected, dried over MgSO_4_, and the solvent removed *in vacuo*. Drying the resulting solid to constant weight using a high vacuum line afforded piroctone as an off-white powder. ^1^H NMR (400 MHz, DMSO) δ 6.19 (d, 1H), 5.93 (d, 1H), 2.59 (dd, 1H), 2.38 (dd, 1H), 2.10 (s, 3H), 2.08 (d, 3H), 2.03 to 1.91 (m, 1H), 1.26 (dd, 1H), 1.08 (dd, 1H), 0.88 (d, 3H), 0.82 (s, 9H).

### β-Galactosidase assays to monitor *mntH-lacZ* promoter activity.

SIP879 (*mntH*:: Mud1(Ap, *lac*) *aroB*) and SIP943 (*mntH*::Mud1(Ap, *lac*) *aroB mntR*) are E. coli K-12 derivatives of MC4100 [*araD139* Δ(*lacIZYA-argF*)*U169 rpsL150 relA1 flhD5301 deoC1 fruA25 rbsR22*] (59) and were kindly provided by Laura Runyen-Janecky. Promoter activity assays were performed as described previously (81). Briefly, bacteria were cultivated in LB broth in sterile cuvettes (1 ml) in the presence or absence of chelant or MnCl_2_ to an *A*_600_ of 0.5. Eighty microliters of culture were transferred to a 96-well microtiter plate followed by addition of 120 μl master mix (60 mM Na_2_HPO_4_, 40 mM NaH_2_PO_4_, 10 mM KCl, 1 mM MgSO_4_, 36 mM β-mercaptoethanol, 166 μl/ml T7 lysozyme, 1.1 mg/ml *o*-nitrophenyl-β-d-galactopyranoside [ONPG], and 6.7% PopCulture reagent obtained from Merck Millipore). This was then transferred to a SPECTROstar Nano absorbance plate reader (BMG Labtech) set to 30°C with shaking at 400 rpm, with absorbance readings taken at 420 and 550 nm every minute for 1 h. Miller units were calculated using the following equation: 1,000 × (*A*_420_ − [1.75 × *A*_550_])/(*T* × *V* × *A*_600_), where *T* is time in minutes, and *V* is volume in milliliters (0.2).

### Keio collection screen.

The duplicate set of 3,985 Keio library mutants, 7,970 strains in total (38), were grown in 200 μl LB media without antibiotic supplementation at 37°C with 27 or 34 μM PO in 96-well microtiter plates for 16 h. A Versette automated liquid handler (Thermo Fisher) was used to dispense media and treatments and inoculate the library. Percentage growth was determined by comparison of *A*_600_, using a SpectraMax plate reader (Molecular Devices), with untreated controls for each strain.

### Determination of cellular metal content.

Different concentrations of chelant were added to 50 ml LB broth in 250-ml acid-washed conical flasks prior to inoculation with 1 × 10^7^ E. coli BW25113 cells. Cultures were grown at 37°C in an orbital shaker at 130 rpm with the aim of inhibiting growth by 10 to 15% during mid-log phase (*A*_650_ of ∼0.3 to 0.4, typically 3 to 4 h of growth). Cell numbers were recorded using a Casy model TT cell counter prior to harvesting. Cells were pelleted by centrifugation (19,000 ×* g*, 25 min) and resuspended in 50 ml wash buffer (0.5 M sorbitol, 10 mM HEPES, pH 7.8) and centrifuged once again at 19,000 ×* g* for 25 min. The cell pellet was then digested in 5 ml 65% nitric acid (Suprapur; Sigma-Aldrich) for a minimum of 16 h. These pellet digests were diluted with 2% nitric acid and 5.89 × 10^−4 ^μM silver standard for ICP (Sigma-Aldrich) in a 1:8:1 ratio. Calibration samples were made using known quantities of metals in nitric acid (ICP multielement standards; CertiPur; Sigma-Aldrich and Merck) diluted in matrix-matched solution. Dilutions and a calibration curve were analyzed using inductively coupled plasma mass spectrometry (ICP-MS; Thermo XSeries 2). Instrument control, analysis, and quantification were obtained using software interface PlasmaLab (Thermo Scientific), and further analysis was conducted in Microsoft Excel. Mean and standard deviation values were determined from triplicate biological analyses.
