# Supplementary material for: Insights into the Antibacterial Mechanism of Action of Chelating Agents by Selective Deprivation of Iron, Manganese, and Zinc
Source: Appl Environ Microbiol. 2022 Jan 25;88(2):e01641-21. doi: 10.1128/AEM.01641-21 (PMC8788741; doi:10.1128/AEM.01641-21)
Supplement: Supplemental file 3 — Tables S1 to S5, Fig. S1 to S10. Download AEM.01641-21-s0001.pdf, PDF file, 5.3 MB [file aem.01641-21-s0001.pdf]

Supplemental Material

Insights into the antibacterial mechanism of action of chelating agents by selective deprivation of iron, manganese and zinc

Joy R. Paterson, Marikka S. Beecroft, Raminder S. Mulla, Deenah Osman, Nancy L. Reeder, Justin A. Caserta, Tessa R. Young, Charles A. Pettigrew, Gareth E. Davies, J.A. Gareth Williams and Gary J. Sharples

TABLE S1 Known metal ion affinities of selected chelants.

Ligand	Mg(II)	Ca(II)	Mn(II)	Fe(II)	Fe(III)	Cu(II)	Zn(II)
BCS		18.8		6.4, 23.2		7.1, 10.8	10.1, 14.9
CAT	1.98	1.7	7.52		18.52	8.09	9.50
CHA					11.24		
DTPA	9.3	10.7	14.31	15.97	28.7	21.5	18.61
DTPMP	6.4	7.11	11.15			19.47	16.45
EDTA	8.83	10.61	13.81	14.27	25.0	18.7	16.44
GLDA	5.2	5.9	7.6		15.35	13.1	11.5
HBED	10.51	9.29	14.78		39.68	22.95	18.95
MGDA	5.8	7.0	8.4	8.1	16.5	13.9	10.9
PO							
TPEN			10.27	14.6		20.6	15.58

The stability or equilibrium constants (K_A) expressed as log K_A, for fully deprotonated chelants, are shown where available; log K_A values at specific pH can be calculated from these absolute stability constants, if the pK_a values of donor atoms are known (1). Values were obtained from the IUPAC Stability Constants Database (2) and were determined at 25°C, I = 0.1 M. The first value for BCS with Cu(II), Zn(II) and Fe(II) refers to ML complex formation; the second refers to ML_2 for Cu(II) and Zn(II) or to ML_3 for Fe(II). BCS with Ca(II) refers to ML_3 formation. Note that formation constants for ML_2 and ML_3 complexes are not directly comparable with ML values. PO is the ethanolammonium salt of piroctone and is predicted to be an effective Fe(III) chelator, based on the donor atom configuration of the piroctone unit being identical to that of the 1,2-HOPO ligand (3).

TABLE S2 Effect of chelants on the total cellular metal content of *E. coli.*

		Metal concentration in atoms per cell at 10-15% growth inhibition					
**Chelant**	**µM**	**Ca (x10^6)**	**Cu (x10^4)**	**Fe (x10^5)**	**Mg (x10^7)**	**Mn (x10^4)**	**Zn (x10^5)**
BCS	0	2.90 ±0.80	0.59 ±0.34	6.88 ±2.30	2.70 ±0.72	3.99 ±1.80	1.80 ±0.71
	3000	4.20 ±0.54	0.48 ±0.32	8.15 ±1.14	3.27 ±0.02	3.98 ±0.91	2.19 ±0.49
CAT	0	4.14 ±0.62	1.53 ±0.57	8.74 ±0.62	3.24 ±0.13	5.53 ±0.19	2.26 ±0.17
	700	3.81 ±0.47	1.00 ±0.15	9.47 ±2.07	3.12 ±0.22	5.74 ±0.43	2.52 ±0.33
	725	3.96 ±0.34	1.08 ±0.20	8.96 ±0.18	3.09 ±0.26	6.10 ±0.29	2.65 ±0.22
	750	3.93 ±0.27	1.18 ±0.14	8.87 ±1.10	3.26 ±0.19	6.14 ±0.38	2.51 ±0.41
	775	4.05 ±0.50	1.10 ±0.05	9.30 ±0.83	3.34 ±0.15	6.10 ±0.52	2.58 ±0.48
	800	3.55 ±0.61	1.44 ±0.16	8.09 ±1.00	3.01 ±0.44	5.30 ±1.35	2.25 ±0.39
	825	3.35 ±0.84	1.23 ±0.20	7.39 ±1.95	2.99 ±0.64	4.92 ±1.76	2.12 ±0.52
CHA	0	4.28 ±0.07	1.29 ±0.07	6.49 ±0.53	2.66 ±0.13	3.70 ±0.26	2.94 ±0.58
	40	4.11 ±0.09	1.49 ±0.20	6.24 ±0.40	2.67 ±0.09	3.76 ±0.07	2.48 ±0.03
	50	3.83 ±0.05	1.41 ±0.09	6.29 ±1.15	2.58 ±0.11	3.48 ±0.23	2.36 ±0.15
DTPA	0	2.85 ±0.10	1.49 ±0.18	6.47 ±0.14	2.11 ±0.10	3.74 ±0.13	2.21 ±0.18
	16	3.17 ±0.13	1.63 ±0.19	5.81 ±0.20	2.14 ±0.28	1.37 ±0.23	1.34 ±0.08
	18	3.27 ±0.23	1.68 ±0.29	4.39 ±1.09	2.09 ±0.33	0.54 ±0.04	1.38 ±0.02
	20	3.43 ±0.25	1.64 ±0.17	5.52 ±1.61	2.89 ±0.35	0.50 ±0.29	1.38 ±0.10
	30	2.84 ±0.10	–	2.40 ±0.01	1.83 ±0.22	0.29 ±0.03	0.83 ±0.08
EDTA	0	3.48 ±0.04	1.40 ±0.33	6.34 ±0.94	3.44 ±0.48	4.62 ±0.29	2.59 ±0.09
	30	3.02 ±0.21	0.77 ±0.04	4.41 ±0.36	3.44 ±0.03	0.35 ±0.04	1.47 ±0.05
	50	2.86 ±0.08	1.41 ±0.45	3.70 ±0.66	3.26 ±0.36	0.35 ±0.02	1.59 ±0.04
	70	2.50 ±0.14	0.91 ±0.10	3.56 ±0.34	3.04 ±0.59	0.37 ±0.06	1.56 ±0.09
GLDA	0	4.16 ±0.08	1.19 ±0.08	8.94 ±0.85	3.22 ±0.12	7.24 ±0.17	2.22 ±0.38
	1000	3.92 ±0.08	1.01 ±0.15	6.78 ±0.26	3.28 ±0.17	2.51 ±0.24	1.11 ±0.10
	5500	3.46 ±0.10	0.78 ±0.06	6.03 ±0.14	3.08 ±0.08	1.06 ±0.02	1.05 ±0.06
	10000	3.24 ±0.11	0.79 ±0.10	6.38 ±0.26	3.00 ±0.20	1.00 ±0.09	1.06 ±0.04
	17500	3.27 ±0.30	1.14 ±0.09	5.44 ±0.55	2.93 ±0.12	0.84 ±0.10	0.99 ±0.10
	25000	3.41 ±0.24	1.32 ±0.10	5.29 ±0.23	3.11 ±0.13	0.89 ±0.04	1.08 ±0.09
MGDA	0	3.93 ±0.16	–	9.91 ±1.25	3.22 ±0.32	5.29 ±0.22	2.59 ±0.38
	2000	3.80 ±0.22	–	9.41 ±1.02	3.25 ±0.44	1.57 ±0.22	1.47 ±0.10
	2250	3.80 ±0.20	–	9.40 ±0.86	3.34 ±0.27	1.44 ±0.19	1.61 ±0.04
	2500	3.86 ±0.10	–	9.65 ±0.46	3.12 ±0.64	1.37 ±0.16	1.51 ±0.14
	2750	3.78 ±0.11	–	9.18 ±0.10	3.39 ±0.34	1.28 ±0.18	1.57 ±0.12
	3000	3.81 ±0.18	–	9.28 ±0.24	3.46 ±0.17	1.23 ±0.05	1.55 ±0.11
	3250	3.74 ±0.27	–	9.05 ±0.52	3.43 ±0.10	1.15 ±0.09	1.50 ±0.05
	3500	3.89 ±0.19	–	8.99 ±0.64	3.51 ±0.25	1.16 ±0.09	1.59 ±0.12
	3750	3.66 ±0.33	–	8.58 ±1.15	3.51 ±0.27	1.12 ±0.15	1.61 ±0.24
	4000	3.96 ±0.26	–	8.97 ±0.77	3.50 ±0.29	1.21 ±0.25	1.64 ±0.27
	4250	3.81 ±0.37	–	8.79 ±1.26	3.53 ±0.35	1.22 ±0.31	1.64 ±0.22
	4500	4.07 ±0.37	–	8.03 ±0.41	3.44 ±0.15	1.10 ±0.16	1.53 ±0.07
	4750	3.48 ±0.22	–	7.48 ±0.13	3.10 ±0.40	0.92 ±0.18	1.39 ±0.14

	5000	3.52 ±0.14	–	7.50 ±0.29	3.16 ±0.48	1.02 ±0.21	1.45 ±0.20
HBED	0	3.79 ±0.15	1.22 ±0.13	9.00 ±0.53	3.41 ±0.11	5.88 ±0.26	2.92 ±0.37
	15	3.84 ±0.18	1.20 ±0.13	4.23 ±0.62	3.26 ±0.38	14.13 ±0.93	3.37 ±1.32
	16	3.76 ±0.10	1.25 ±0.04	3.86 ±0.53	3.30 ±0.04	14.87 ±0.18	2.44 ±0.17
	17	3.75 ±0.19	1.15 ±0.13	3.77 ±0.49	2.99 ±0.34	15.26 ±1.10	2.54 ±0.29
	18	3.76 ±0.19	1.26 ±0.14	3.68 ±0.41	3.17 ±0.19	16.30 ±1.48	2.44 ±0.21
	19	4.03 ±0.14	1.60 ±0.57	3.45 ±0.29	3.32 ±0.19	17.11 ±0.31	2.60 ±0.32
	20	3.94 ±0.02	1.17 ±0.13	3.30 ±0.19	3.33 ±0.23	17.60 ±0.51	2.60 ±0.27
DTPMP	0	3.69 ±0.12	–	6.21 ±0.14	3.69 ±0.18	5.21 ±0.21	3.20 ±0.14
	10	3.54 ±0.13	–	2.35 ±0.13	3.54 ±0.18	14.36 ±0.30	3.30 ±0.24
PO	0	3.73 ±0.04	1.78 ±0.20	8.35 ±0.65	2.85 ±0.27	3.87 ±0.91	2.52 ±0.70
	2	3.62 ±0.17	–	7.88 ±0.32	2.72 ±0.27	3.95 ±0.14	2.12 ±0.16
	4	3.53 ±0.04	–	7.52 ±0.35	2.52 ±0.58	3.96 ±0.07	2.21 ±0.30
	5	3.33 ±0.49	–	8.40 ±2.11	2.65 ±0.18	3.30 ±1.47	2.15 ±0.54
	6	3.39 ±0.47	–	6.98 ±0.70	2.42 ±0.62	2.71 ±1.82	1.77 ±0.39
	8	3.56 ±0.57	–	7.47 ±0.91	2.09 ±0.93	3.70 ±1.90	2.17 ±0.59
	10	2.77 ±1.54	–	7.39 ±0.05	2.17 ±0.40	3.84 ±2.09	2.16 ±0.58
	12	3.78 ±0.22	–	5.66 ±1.07	2.11 ±0.80	7.66 ±1.03	2.27 ±0.28
	14	3.63 ±0.12	–	3.71 ±0.69	2.38 ±0.56	12.05 ±1.12	2.22 ±0.24
	16	3.48 ±0.12	–	3.39 ±1.07	2.49 ±0.62	14.28 ±1.13	2.22 ±0.44
	18	3.46 ±0.14	–	3.28 ±0.46	1.94 ±0.80	16.06 ±1.70	2.43 ±0.41
	20	3.94 ±0.46	1.53 ±0.11	3.83 ±1.22	2.52 ±0.72	17.60 ±3.80	3.37 ±1.12
TPEN	0	4.40 ±0.23	1.30 ±0.94	8.93 ±0.15	3.01 ±0.17	5.60 ±0.44	2.36 ±0.14
	300	4.49 ±0.11	1.77 ±0.54	9.08 ±0.75	3.21 ±0.04	5.27 ±0.11	1.61 ±0.09
	305	4.69 ±0.40	1.36 ±0.96	9.34 ±0.22	3.12 ±0.44	5.29 ±0.58	1.72 ±0.02
	310	4.66 ±0.22	1.71 ±0.41	8.85 ±0.47	3.31 ±0.20	5.00 ±0.79	1.69 ±0.03
	315	4.57 ±0.27	1.70 ±0.43	10.22 ±0.71	3.28 ±0.25	4.54 ±0.65	1.58 ±0.08
	320	4.36 ±0.16	1.90 ±0.03	9.59 ±0.34	3.11 ±0.22	3.95 ±0.38	1.50 ±0.11

Total cellular metal concentration was monitored for *E. coli* with 10-15% growth inhibition over a range of chelant concentrations. *E. coli* BW25113 was grown in 50 ml of LB media at 37°C in a shaking incubator in the presence or absence of chelant to mid log-phase (A_{650nm} of 0.3-0.4, 3-4 hours). Data are the mean and standard deviation of three independent experiments. Red text indicates a reduction in total cellular metal content of 1.5-2 fold (■), 2-3 fold (■) or >3 fold (■), whereas blue text indicates an increase in metal content of 2-3 fold (■) or >3 fold (■). Boxes shaded in grey with copper should be interpreted with caution as the values were below the usual cut-off for the calibration curve used to evaluate accuracy of concentrations. The standard used 0, 0.1, 0.2, 0.5, 1, 2, 5, 10, 20, 50, 100, 200, 500 and 1000 ppb standard solutions prepared from a multi-element stock solution containing calcium, magnesium, cobalt, copper, iron, manganese, nickel and zinc, all at 1000 ppm, to generate the calibration curve.

TABLE S3 Effect of selected chelants in combination on the total cellular metal content of *E. coli.*

		Fold-change relative to control				
**DTPA/PO combinations**	**inhibition**	**calcium**	**iron**	**magnesium**	**manganese**	**zinc**
16 µM DTPA	12%	1.0 ±0.23	0.8 ±0.12	1.0 ±0.15	0.4 ±0.12	0.8 ±0.15
14 µM PO	9%	1.0 ±0.05	0.6±0.12	1.0 ±0.24	2.7 ±0.62	0.9 ±0.13
6 µM DTPA + 14 µM PO	23%	1.1 ±0.03	0.5 ±0.11	1.1 ±0.24	2.8 ±0.25	0.9 ±0.14
8 µM DTPA + 14 µM PO	33%	0.9 ±0.02	0.5 ±0.04	0.9 ±0.21	2.9 ±0.13	0.8 ±0.03
10 µM DTPA + 14 µM PO	14%	1.1 ±0.02	0.6 ±0.20	0.6 ±0.02	2.8 ±0.61	0.7 ±0.04
**DTPMP/PO combinations**	**inhibition**	**calcium**	**iron**	**magnesium**	**manganese**	**zinc**
10 µM DTPMP	7%	1.0 ±0.11	0.5 ±0.10	1.0 ±0.07	2.5 ±0.57	0.7 ±0.05
10 µM DTPMP + 12 µM PO	6%	1.0 ±0.06	0.4 ±0.11	1.0 ±0.01	2.7 ±0.26	0.7 ±0.05
10 µM DTPMP + 16 µM PO	14%	1.1 ±0.26	0.4 ±0.09	1.0 ±0.04	2.8 ±0.09	0.7 ±0.13
15 µM PO	13%	1.1 ±0.24	0.9 ±0.32	1.0 ±0.13	1.6 ±0.22	0.9 ±0.02
15 µM PO + 5 µM DTPMP	27%	1.1 ±0.21	0.5 ±0.25	1.1 ±0.03	2.6 ±0.11	0.7 ±0.01
15 µM PO + 10 µM DTPMP	34%	0.9 ±0.19	0.5 ±0.33	1.0 ±0.04	3.2 ±0.57	0.6 ±0.03

E. coli BW25113 cells were grown in 50 ml of LB media to mid-log phase (absorbance at 650nm of 0.2-0.3) in a shaking incubator at 37°C. Data are the mean and standard deviation of at least 3 independent experiments. The mean percentage growth inhibition is indicated. Red shading indicates a reduction in metal content of ≤0.7-fold, whereas blue shading indicates an increase in metal content of >1.5-fold.

TABLE S4 Keio collection mutants with improved growth following treatment with PO.

		% growth	
**Mutant**	**Gene function**	**27 µM PO**	**34 µM PO**
mutY	DNA adenine glycosylase, base excision repair, Fe-S cluster protein	227	178
rbsD	Peptidoglycan hydrolase, flagellar biosynthesis	197	161
mobB	Molybdenum (Mo) cofactor biosynthesis	189	142
yiiM	Hydroxylaminopurine resistance, DNA damage tolerance, binds Mo	170	125
yicM	Purine ribonucleoside exporter, MFS transporter	168	129
yqhC	DNA binding transcriptional activator, possible aldehyde response	163	124
yiiF	Unknown	157	125
gldA	Glycerol dehydrogenase; glycerol metabolism	155	117
yrfD	DNA utilization; involved in DNA uptake and use as a carbon source	154	116
yhiM	Inner membrane protein involved in acid tolerance	150	111
yieP	DNA binding transcriptional regulator, possible membrane integrity role	147	114
yjdC	DNA binding transcriptional regulator, possible thiol stress response	146	108
yjfN	Protease activator in periplasm, activator of DegP proteolysis	146	110
yjjM	DNA binding transcriptional regulator, L-galactonate metabolism	139	105
yjgB	Aldehyde reductase, lipid metabolism	137	103
yjgF	Enamine/imine deaminase, amino acid metabolism	136	103
trxA	Thioredoxin, redox homeostasis	135	100
flgJ	Peptidoglycan hydrolase, flagellar biosynthesis	132/109	104/91
tnaB	Tryptophan symporter, tryptophan metabolism	128	97
flgK	Flagellum hook-filament junction protein, flagellar biosynthesis	127	99
glcF	Glycolate dehydrogenase, glycolate metabolism, Fe-S cluster protein	126	94
nuoC	Quinone oxidoreductase, interacts with flagellum motor	126/103	112/95
yghO	DNA binding transcriptional regulator, linked to biofilm formation	126	94
recQ	DNA helicase, DNA replication, recombination and repair	125	93
flgG	Flagellum basal-body rod protein, flagellar biosynthesis	124/113	97/97
flgI	Flagellum P-ring protein, flagellar biosynthesis	124/118	101/97
fliN	Flagellum motor switch protein, flagellar biosynthesis	124/111	104/102
flgF	Flagellum basal-body rod protein, flagellar biosynthesis	123/115	101/99
elaD	Protease	123	90
yjhT	N-acetylneuraminate mutarotase, carbohydrate metabolism	123	94
yjiA	Guanosine triphosphatase	123	94
ymfS	Putative tail fibre assembly protein from prophage e14	122	90
ylbH	Putative RHS domain-containing protein	121	91
ymdE	Unknown	120	91
fliI	Flagellum-specific ATP synthase, flagellar biosynthesis	120	99
ymfP	Unknown from prophage e14	120	87
flhD	DNA binding transcriptional dual regulator of flagellar biosynthesis	119/105	101/98
flgL	Flagellar hook-filament junction protein, flagellar biosynthesis	119	104
yedQ	Putative diguanylate cyclase, cellulose metabolism and flagellum	118	91
flgB	Flagellar basal-body rod protein, flagellar biosynthesis	118/114	100/99
flgC	Flagellar basal-body rod protein, flagellar biosynthesis	118	99
prfB	peptide chain release factor, translation termination	117	87
flgE	Flagellar hook protein, flagellar biosynthesis	116	97
dnaK	Chaperone, protein folding	116/111	114/104
fliG	Flagellar motor switch protein, flagellar biosynthesis	115	91

The 45 mutants with most improved growth at 27 μM PO are listed alongside those showing enhanced growth at 34 μM PO. The percentage growth represents the relative proportion of *E. coli* growth in the control with and without PO treatment in LB media at 37°C. Experiments with PO at each concentration were performed with a duplicate set of Keio collection mutants and two values are included for those mutants that appeared in the top 200 mutants showing the best growth in the presence of PO. Greater significance, in terms of likelihood of genuine growth improvement, is attached to those mutants that appear at both concentrations of PO, and furthermore if both duplicates show similarly improved growth at a single concentration. The complete data set can be found in Supporting Information Data File S2.

TABLE S5 Chelating agents used in this study.

Synonym	Formal Name	CAS Number	Source
BCS	Bathocuproine disulphonic acid (disodium salt)	52698-84-7	Sigma-Aldrich
CAT	Catechol, 1,2-dihydroxybenzene	120-80-9	Sigma-Aldrich
CHA	Caprylhydroxamic acid, octanohydroxamic acid	7377-03-9	Fluorochem
DTPA	Diethylenetriaminepentaacetic acid	67-43-6	Sigma-Aldrich
DTPMP	Diethylenetriaminepentakis(methylphosphonic acid)	15827-60-8	Sigma-Aldrich
EDTA	Ethylenediaminetetraacetic acid	60-00-4	Melford
GLDA	N,N-bis(carboxymethyl)-L-glutamic acid (tetrasodium salt)	51981-21-6	Biosynth Carbosynth
HBED	N,N'-di(2-hydroxybenzyl)ethylene diamine-N,N'-diacetic acid (monohydrochloride hydrate)	35369-53-0	Strem Chemicals
MGDA	Methylglycinediacetic acid, N-(1-carboxylatoethyl)iminodiacetate (trisodium hydrate)	164462-16-2	TCI
PO	Piroctone olamine, 1-hydroxy-4-methyl-6-(2,4,4-trimethylpentyl)-2(1H)-pyridone ethanolammonium salt	68890-66-4	Combi-Blocks
TPEN	N,N,N',N'-tetrakis(2-pyridinylmethyl)-1,2-ethanediamine	16858-02-9	Cayman Chemical

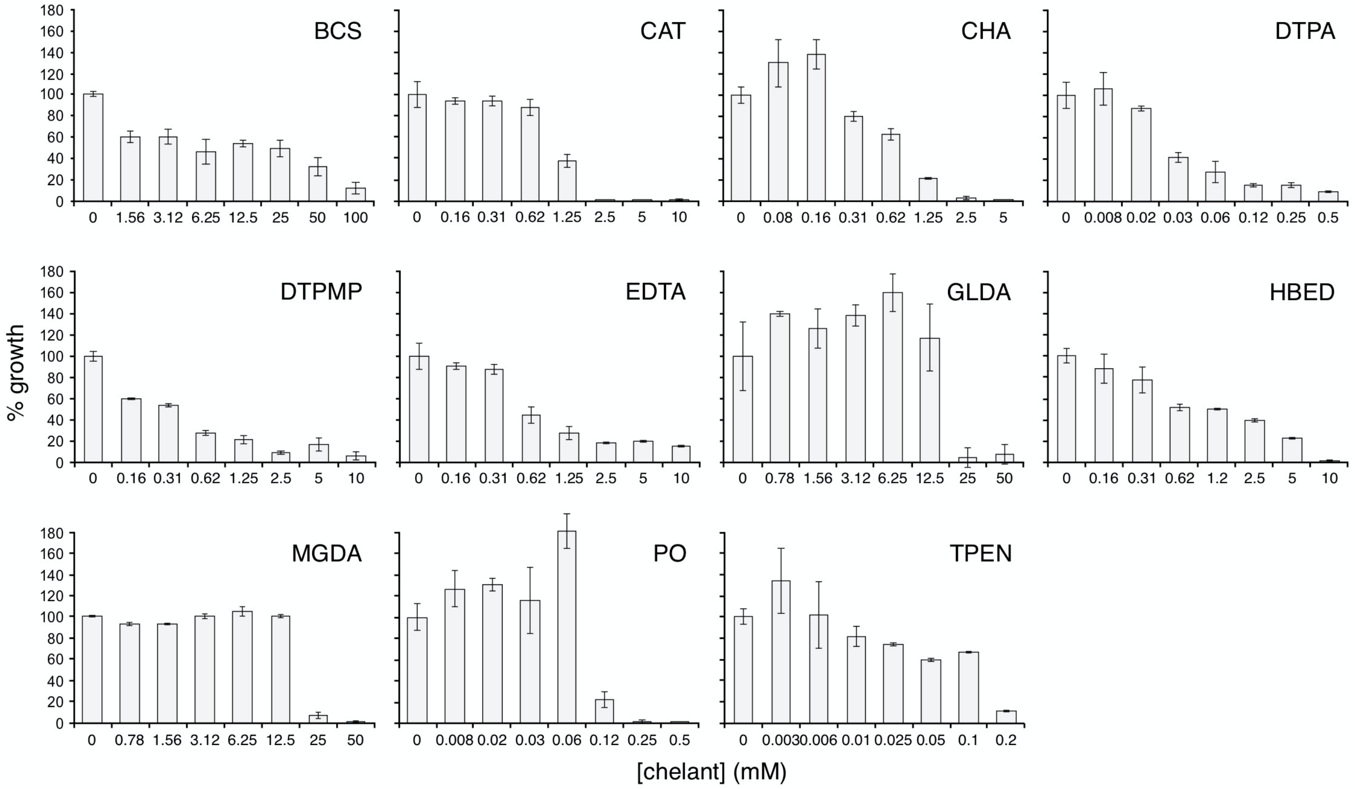

FIG S1 Effect of chelants on *E. coli* growth in MOPS-minimal medium. Bacteria were cultivated in MOPS-minimal media supplemented with 0.2% glucose and mixed with appropriate 2-fold dilutions of each chelant and incubated with shaking for 16 h at 37°C. Results are the mean and standard deviation of an independent experiment performed in triplicate; a further three independent experiments performed in triplicate yielded similar results.

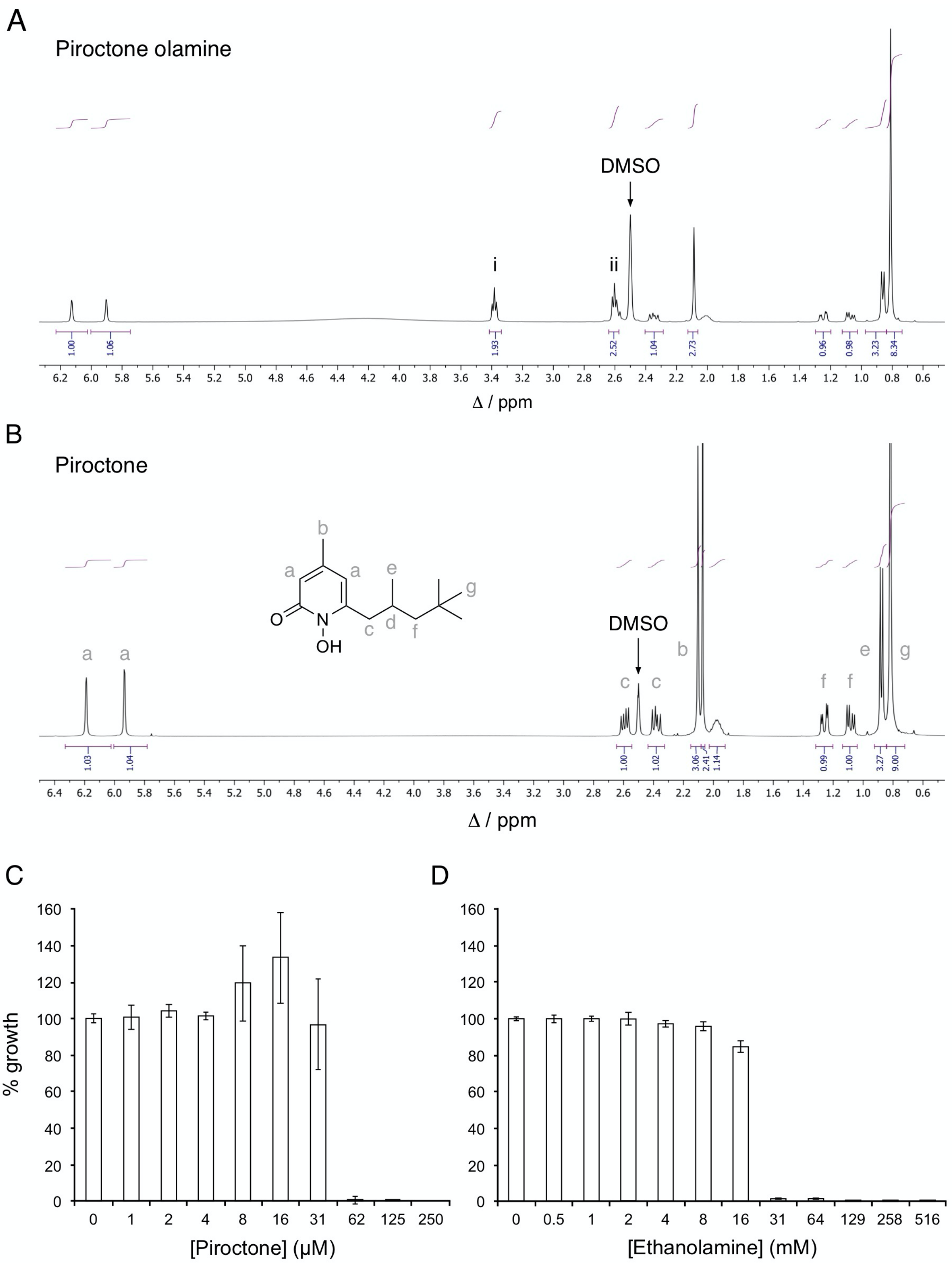

FIG S2 NMR spectra demonstrating the separation of piroctone from piroctone olamine and effect of piroctone and ethanolamine on bacterial growth. (A) ^{1}H NMR spectrum in d6-DMSO of commercially obtained piroctone olamine used in this study with the CH_2 resonances of ethanolamine labelled at i) 3.4 and ii) 2.6 ppm. (B) The corresponding spectrum of the isolated piroctone confirming the removal of ethanolamine. *E. coli* growth inhibition by (C) piroctone and (D) ethanolamine. Bacteria were cultivated in LB media at 37°C, diluted and mixed with appropriate concentrations of piroctone or ethanolamine and incubated with shaking for 16 h.

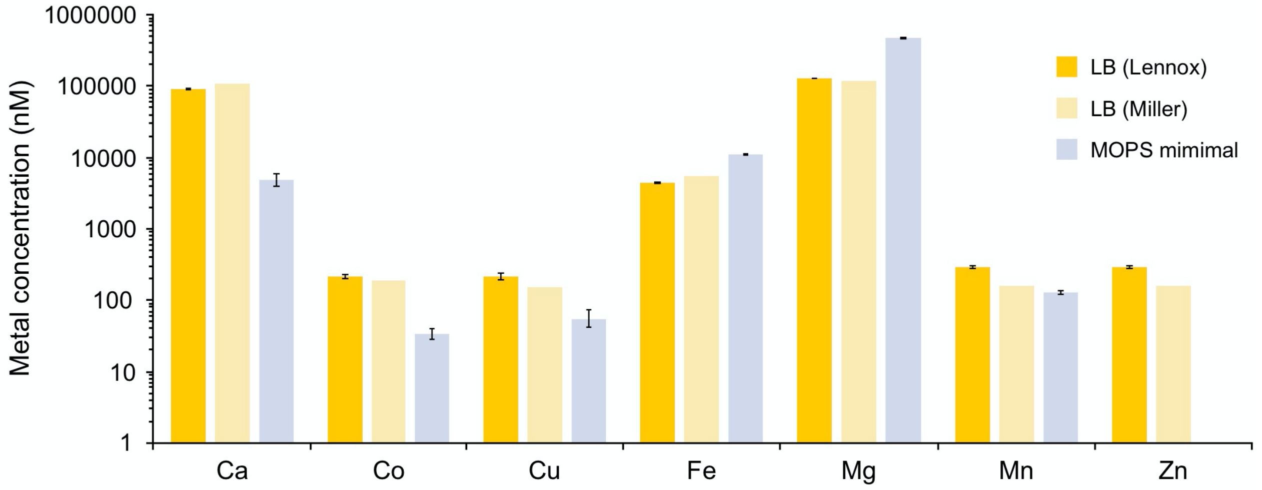

FIG S3 Concentration of metals in LB (Lennox) broth and MOPS minimal media used in this study. Metal concentration was determined by ICP-MS on diluted samples at 1:10 and 1:100 dilutions. Experiments are the mean and standard deviation of three independently prepared samples of media. The concentration of nickel was below the limit of detection in LB and MOPS-minimal media; this was also the case for zinc in the latter. Previously published metal analysis (4, 5) of LB (Miller) broth, provided by Caryn Outten, is included to facilitate comparisons with LB (Lennox).

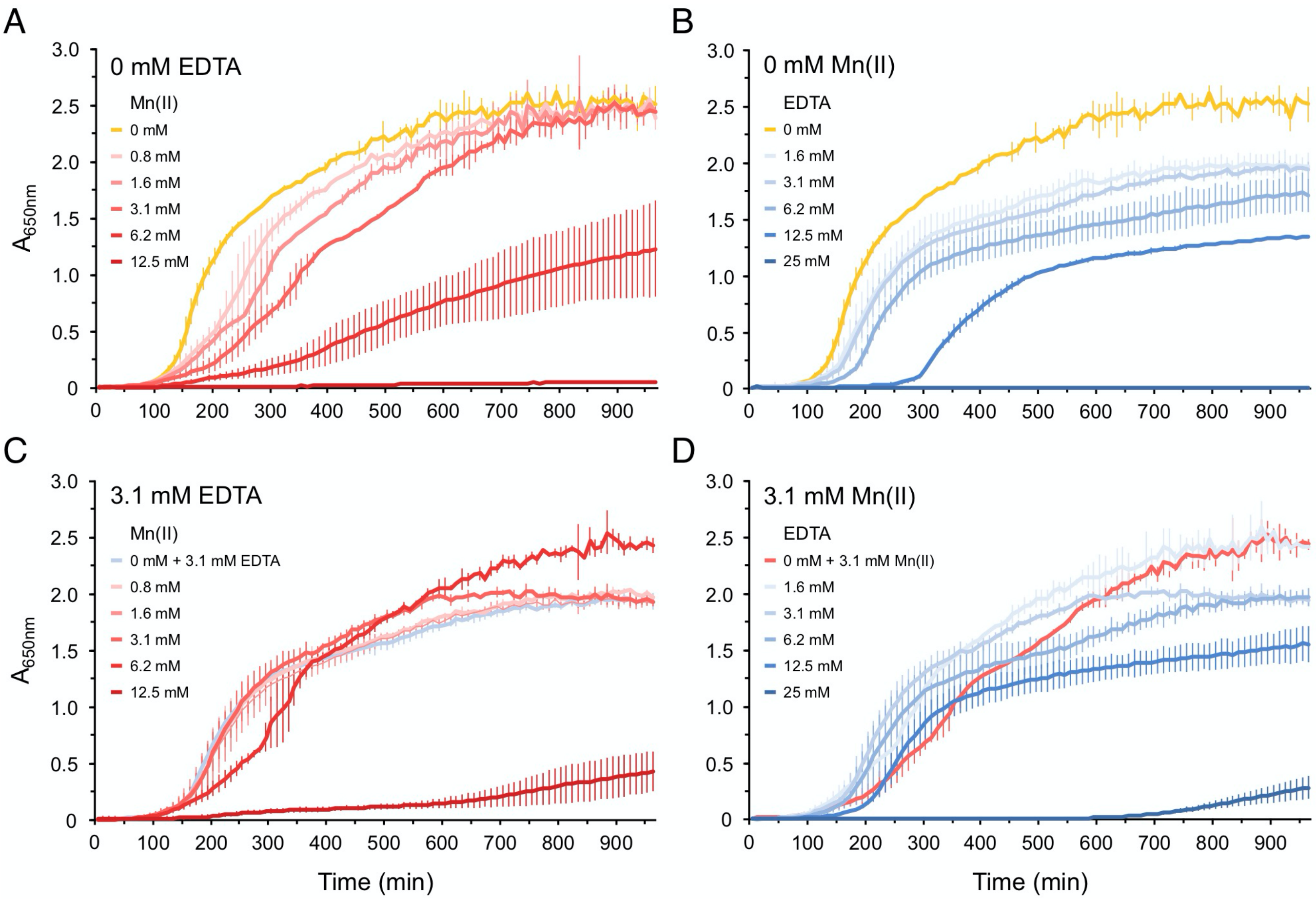

FIG S4 Effect of manganese supplementation on *E. coli* growth inhibition by EDTA. Bacteria were cultivated at 37°C in LB supplemented with $MnCl_2$ (red) or EDTA (blue) at the concentrations indicated, with shaking in a plate reader and growth monitored by absorbance at 650 nm (A_{650nm}). The growth curves are the mean of two independent experiments, each performed in triplicate.

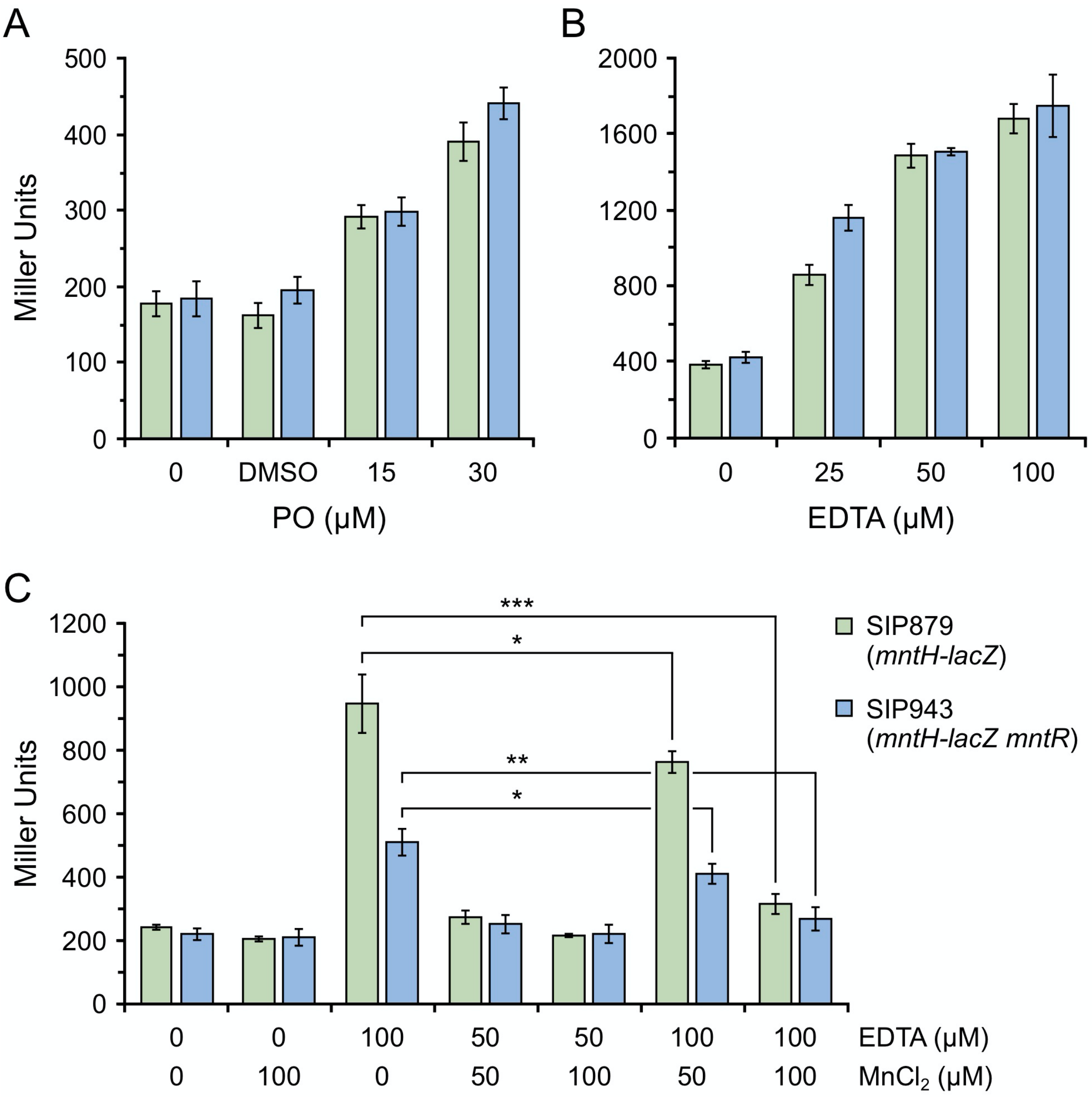

FIG S5 Effect of PO, EDTA and manganese chloride on *mntH-lacZ* expression. Response of *E. coli mntH-lacZ mntR*[+] (SIP879) and *mntH-lacZ mntR* (SIP943) to PO (A), EDTA (B) and EDTA supplemented with $MnCl_2$ (C). Miller Units were calculated from absorbance values obtained 40 minutes after addition of the β-galactosidase reaction mix in all cases and according to the equation provided in the Materials and Methods. Cells were grown in LB media with chelants and/or $MnCl_2$ included at the outset. Data are the mean and standard deviation of 3 independent biological replicates with one-way ANOVA used to compare each chelant concentration with the untreated control: PO, $P < 0.01$ (A) and EDTA, $P < 0.001$ (B) or as indicated between relevant data sets, *P < 0.05, ***P* < 0.01 and ****P* < 0.001 (C). Levels of expression varied between different reaction preparations so independent experiments were performed in the same 96-well microtitre plate where possible. PO at 30 µM resulted in some inhibition of bacterial growth.

CAT	CHA	DTPA	DTPMP	EDTA	GLDA	HBED	MGDA	PO	TPEN	
1.00/1.16	1.16/1.66	0.56/0.87	1.58/1.83	1.00/1.16	0.91/1.16	1.16/1.33	1.16/1.16	1.16/1.33	1.02/2.33	BCS
	1.08/1.10	0.28/0.37	0.34/0.63	0.63/0.67	0.43/0.59	0.94/0.99	0.48/0.68	1.09/1.22	0.53/0.67	CAT
		0.27/0.62	0.41/0.67	0.50/0.66	0.81/1.12	0.87/0.93	0.99/1.05	1.05/1.18	0.57/0.61	CHA
			0.62/0.65	1.50/1.55	0.32/0.41	0.37/0.49	0.41/0.73	0.32/0.67	0.67/1.11	DTPA
				1.43/2.10	1.47/1.50	0.77/0.88	1.85/1.85	0.41/0.42	0.52/0.56	DTPMP
					0.61/0.88	0.80/0.94	0.65/0.65	0.37/0.42	1.03/1.16	EDTA
						0.93/1.27	0.81/0.90	1.04/1.21	1.49/1.49	GLDA
							1.10/1.39	0.92/1.10	0.55/0.74	HBED
								1.10/1.21	1.10/1.38	MGDA
									0.88/0.93	PO

Synergistic ≤0.5

Indifferent >0.5-≤4.0

FIG S6 Chelant combinations analysed by the checkerboard assay using a mean FIC methodology. FIC indices from two independent experiments were calculated from the data presented in Supplementary Data File S1. In eight instances different outcomes were found between the two experiments and these have been conservatively assigned as indifferent. MICs were determined for individual chelants as before with FIC values averaged from the boundaries between growth and <10% *E. coli* growth of chelants in combination.

Synergistic	
BCS	DTPA
CAT	DTPA
CAT	DTPMP
CAT	EDTA
CAT	GLDA
CAT	MGDA
CAT	TPEN
CHA	DTPA
CHA	DTPMP
CHA	EDTA
CHA	GLDA
CHA	HBED
CHA	TPEN
DTPA	DTPMP
DTPA	GLDA
DTPA	HBED
DTPA	MGDA
DTPA	PO
DTPA	TPEN
DTPMP	HBED
DTPMP	PO
DTPMP	TPEN
EDTA	GLDA
EDTA	MGDA
EDTA	PO
GLDA	MGDA
HBED	TPEN
PO	TPEN

Indifferent	
BCS	CAT
BCS	CHA
BCS	DTPMP
BCS	EDTA
BCS	GLDA
BCS	HBED
BCS	MGDA
BCS	PO
CAT	CHA
CAT	HBED
CAT	PO
CHA	MGDA
CHA	PO
DTPA	EDTA
DTPMP	EDTA
DTPMP	GLDA
DTPMP	MGDA
EDTA	HBED
EDTA	TPEN
GLDA	HBED
GLDA	PO
GLDA	TPEN
HBED	MGDA
HBED	PO
MGDA	PO
MGDA	TPEN

Antagonistic	
BCS	TPEN

Key: metal effects

No change	↓Fe ↑Mn
↓Mn ↓Fe ↓Zn	↓Zn ↓Mn

FIG S7 Comparison of synergistic, indifferent and antagonistic combinations with the four chelant categories defined by their distinct effects on total cellular metal concentration. Bars are colour-coded according to their effect on metals as indicated in the key.

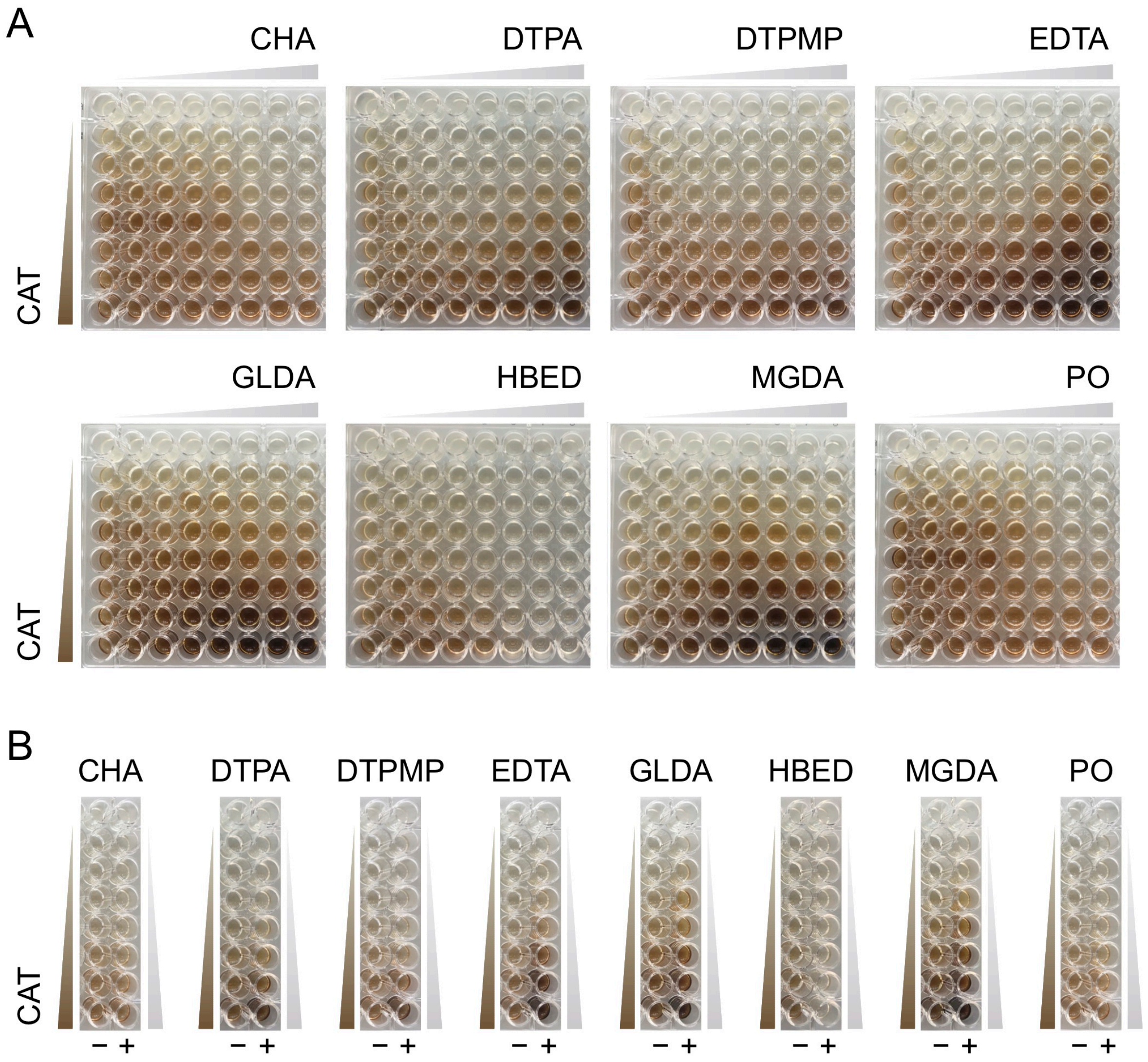

FIG S8 Effect of CAT-Fe(III) complex formation in the presence of other chelants. (A) Examples of checkerboards of CAT with selected chelants in the presence of *E. coli*. Two-fold serial dilutions of CAT (0.5-30 mM) from top to bottom of each 96-well plate. Similar dilutions of other chelants, as indicated, were applied from left to right to create the checkerboard. The first column contains CAT alone while the first row is the appropriate chelant on its own: CHA (0.04-2.5 mM), DTPA (0.4-25 mM), DTPMP (0.4-22.5 mM), EDTA (0.8-50 mM), GLDA (2-125 mM), HBED (0.2-10 mM), MGDA (2-125 mM) and PO (0.002-0.15 mM). (B) Effect of chelants on CAT-Fe(III) complexation in the absence of *E. coli*. CAT dilutions alone (–) or CAT in combination with another chelant (+) are shown. Chelant concentrations are the same as those in (a). All experiments were performed in LB broth and incubated at 37°C for 16 h.

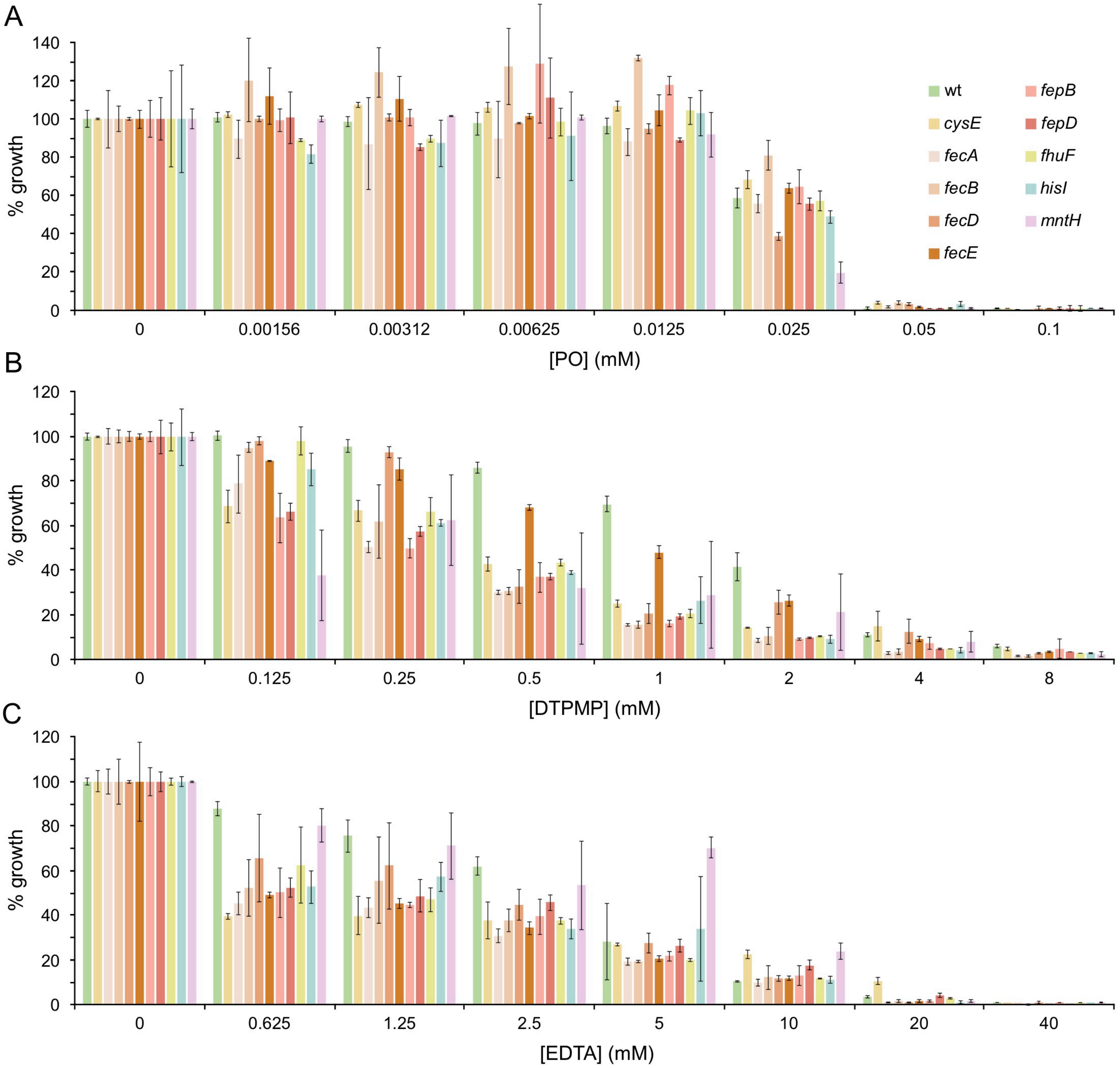

FIG S9 Selected *E. coli* mutant sensitivity to (a) PO, (b) DTPMP and (c) EDTA. Bacteria were incubated with a two-fold serial dilution of each chelant in 100 µl of LB media and incubated with shaking for 16 h at 37°C. Absorbance at 600 nm was monitored and percentage growth determined. Data represent the mean and standard deviation of a single independent experiment performed in triplicate. A second independent experiment performed in triplicate produced a similar pattern of susceptibility. The *E. coli* FhuABCDEF system can import the hydroxamate-type siderophore ferrichrome-iron(III) complexes made by other microbes, while the FecABCDE system takes up ferric iron, Fe(III), in complex with citrate (6). In response to manganese starvation, *E. coli* mediates Mn(II) import using MntH (7, 8), an evolutionary-conserved, NRAMP-family, proton-driven transporter equivalent to the one used by phagocytes to remove divalent cations from the phagolysosome (9, 10).

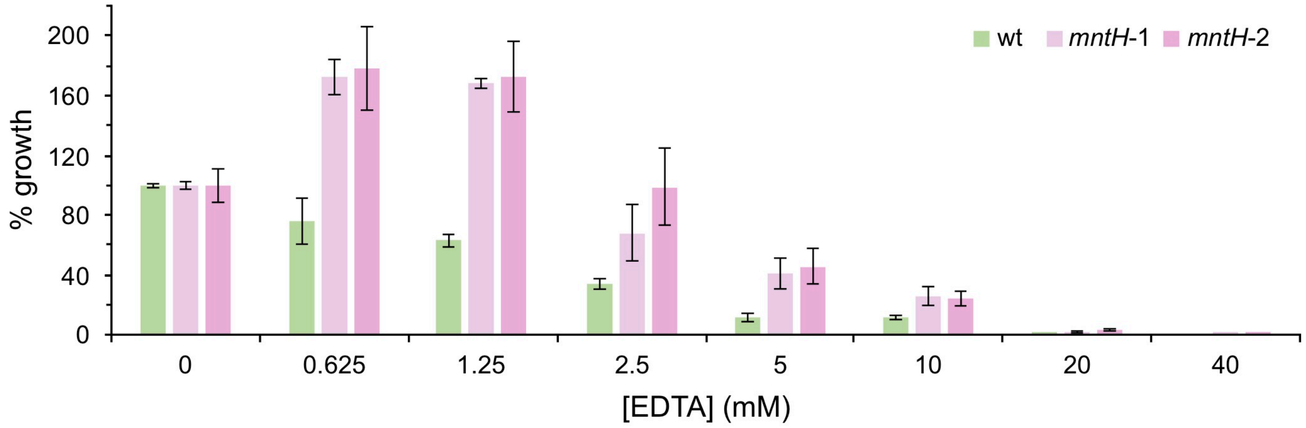

FIG S10 Sensitivity of *E. coli mntH* to EDTA. Bacteria were incubated with a two-fold serial dilution of chelant in 100 µl of LB media and incubated with shaking for 16 h at 37°C. Absorbance at 600 nm was monitored and percentage growth determined. Samples of wt (BW25113) and *mntH*-1 and *mntH*-2 represent the mean and standard deviation of independent experiments performed in triplicate.

References

1. Xiao Z, Wedd AG. 2010. The challenges of determining metal-protein affinities. Nat Prod Rep 27:768-789.
2. Pettit LD. 2009. The IUPAC stability constants database. Chemistry International - Newsmagazine for IUPAC 28:14-15, https://doi.org/10.1515/ci.2006.28.5.14.
3. Li YJ, Martell AE. 1993. Potentiometric and spectrophotometric determination of stabilities of the 1-hydroxy-2 pyridinone complexes of trivalent and divalent metal ions. Inorg Chim Acta 214:103-111.
4. Outten CE, O'Halloran TV. 2001. Femtomolar sensitivity of metalloregulatory proteins controlling zinc homeostasis. Science 292:2488-2492.
5. Outten CE. 2001. Mechanistic studies of metalloregulatory proteins controlling metal ion homeostasis in *Escherichia coli*. Doctoral dissertation. Northwestern University.
6. Miethke M. 2013. Molecular strategies of microbial iron assimilation: from high-affinity complexes to cofactor assembly systems. Metallomics 5:15-28.
7. Makui H, Roig E, Cole ST, Helmann JD, Gros P, Cellier MF. 2000. Identification of the *Escherichia coli* K-12 Nramp orthologue (MntH) as a selective divalent metal ion transporter. Mol Microbiol 35:1065-1078.
8. Kehres DG, Zaharik ML, Finlay BB, Maguire ME. 2000. The NRAMP proteins of *Salmonella typhimurium* and *Escherichia coli* are selective manganese transporters involved in the response to reactive oxygen. Mol Microbiol 36:1085-1100.
9. Juttukonda LJ, Skaar EP. 2015. Manganese homeostasis and utilization in pathogenic bacteria. Mol Microbiol 97:216-228.
10. Kelliher JL, Kehl-Fie TE. 2016. Competition for manganese at the host-pathogen interface. Prog Mol Biol Transl Sci 142:1-25.
